# Modulation of Al Sites in MWW Zeolites with Enhanced Catalytic Performance by Dual Organic Structure-Directing Agents

**DOI:** 10.1021/cbe.5c00016

**Published:** 2025-04-10

**Authors:** Chuang Liu, Guodong Qi, Yudan Gong, Darui Wang, Wenhua Fu, Fang Liu, Jun Xu, Dianhua Liu, Zhendong Wang, Weimin Yang

**Affiliations:** † School of Chemical Engineering, 47860 East China University of Science and Technology, Shanghai 200237, China; ‡ State Key Laboratory of Green Chemical Engineering and Industrial Catalysis, SINOPEC Shanghai Research Institute of Petrochemical Technology Co., Ltd., Shanghai 201208, China; § National Center for Magnetic Resonance in Wuhan, State Key Laboratory of Magnetic Resonance Spectroscopy and Imaging, Innovation Academy for Precision Measurement Science and Technology, Chinese Academy of Sciences, Wuhan 430071, China

**Keywords:** MWW, Zeolite, Al sites, TMAdaOH, Cyclohexylamine, OSDAs

## Abstract

MWW zeolites exhibit a distinctive combination of both 10-*ring* and 12-*ring* features, making them highly versatile in catalytic applications. When dealing with bulk molecules, the acid sites located on the external surface often serve as the primary active sites, and thus, the distribution of Al sites significantly impacts the active acidity and catalytic performance. In this study, a precise modulation of Al distribution within MWW zeolites was investigated using commercially available N,N,N-trimethyl-1-adamantylammonium hydroxide (TMAdaOH) and cyclohexylamine as organic structure-directing agents (OSDAs). The effects of both OSDAs, along with the synthesis temperature, duration and Na^+^, on the formation of the MWW framework were systematically examined. Advanced solid-state NMR characterization addressed the correlation between Al and organic species. The presence of TMAdaOH showed significant influence on the distributions of cyclohexylamine and T_2_ Al sites as well as the benzene transport rate, resulting in MWW zeolites with enriched external surface accessible active acid sites. Owing to these properties, the MWW zeolites exhibited superior catalytic performance compared to conventional MCM-22 zeolites in the cracking of TiPB and alkylation of benzene with cyclohexene. This study highlights the successful synthesis of MWW zeolites using small amounts of low-toxicity OSDAs, offering a scalable and economical approach for the production of zeolites with enhanced catalytic properties for industrial applications.

## Graphical abstract

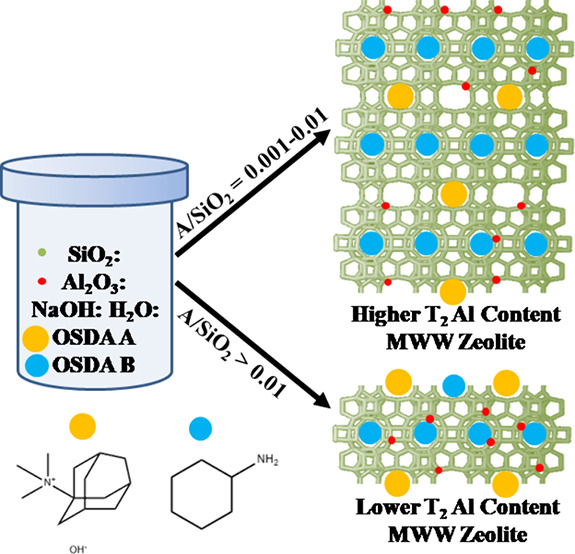

## 1 Introduction

Zeolites are vital inorganic crystalline porous materials with remarkable shape selectivity, exceptional chemical and (hydro)­thermal stability and tunable framework compositions.^1,2^ These properties establish zeolites as indispensable materials for heterogeneous catalysis and adsorption, with extensive applications in the petrochemical, coal chemical and fine chemical industries. MWW-type zeolites with layered structures, are consisted of thin layers with thickness of ∼2.5 nm usually oriented perpendicular to the *c*-axis.^3,4^ There are many members of the MWW zeolite family, including MCM-22,^3^ MCM-36,^5^ MCM-49,^6^ MCM-56,^7^ UCB-1,^8^ UZM-8,^9^ ITQ-1,^10^ ITQ-2,^4^ SSZ-25,^11^ ECNU-5,^12^ ECNU-7,^13^ ECNU-10,^14^ SL-MWW,^15^ SCM-1 and SCM-6,^16^ prepared through hydrothermal synthesis and post-treatment methods.^17–20^ These zeolites feature two independent and disconnected pore channel systems (Scheme 1): the 10-*ring* two-dimensional (2D) sinusoidal channels within the layers, the interlayer 12-*ring* supercages which are accessible from the exterior via 10-*ring* channels. Meanwhile, the 12-*ring* cup-shaped semi-supercages (surface pockets) with a depth of ∼0.70 nm are present on the external surface.

**Scheme 1:**
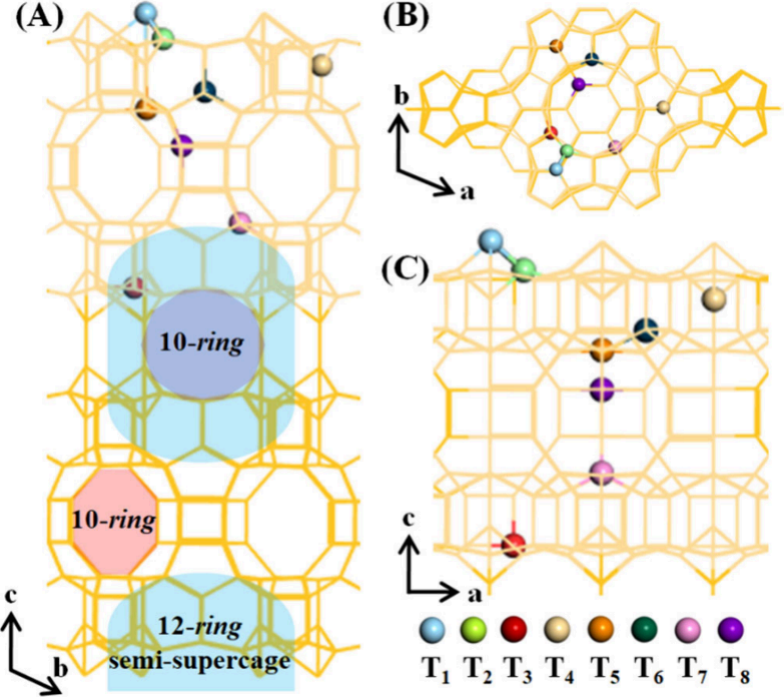
MWW Topology and Channel Structure^a^

a (A) 12-*ring* semi-supercages on the external surface (light blue), 12-*ring* supercages which are accessible from the exterior *via* 10-*ring* channels (light violet) and 10-*ring* sinusoidal channels within the layers (light red), (B) top view along the *c*-axis and (C) side view along the *b*-axis revealing the spatial arrangement of eight distinct T sites.

The unique structure enables MWW zeolites to exhibit both 10-*ring* and 12-*ring* features, endowing them with distinctive acid properties and exceptional catalytic versatility in a variety of reactions, including alkylation of benzene, catalytic cracking of heavy aromatic and isomerization of olefin.^20–23^ Different from surface acid sites on many other zeolites, the acid sites in the surface pockets of MWW-type zeolite exhibit strong acidity, similar to those within the micropores.^24^ Thus, in the alkylation of benzene with olefins, especially with bulky olefins, it is generally accepted that increased external surface area is an effective way to enhance catalytic performance.^25,26^ The attempt to synthesize ultrathin MWW zeolites with significantly large external surfaces has emerged as one of the most interesting research topics in this field.^20^ Through post-treatment strategies, delaminated MWW zeolites were fabricated by employing a swelling process followed by layer exfoliation (yielding zeolites such as ITQ-2^4^ and UCB-1 ^8^) or by pillaring (producing such as MCM-36^5^ and ECNU-7 ^13^) of MCM-22P layered precursors in the presence of surfactant.^27^ The delaminated ITQ-2 demonstrates an exceptionally high external surface area with the thickness of a single layer (2.5 nm).^4^ However, the post-treatment modification procedure is quite intricate and typically carried out under harsh alkaline or acidic conditions, which would lead to partial degradation of the structure, resulting in low crystallinity and yield.^20,26^

Thus, one-step strategies for the direct syntheses of MWW zeolites were developed. By employing “bifunctional” systems constructed by dual amines or with the presences of specifically designed organic structure-directing agents (OSDAs), ultrathin MWW zeolites such as SCM-1,^16^ DS-ITQ-2,^28^ MIT-1 ^29^ and UJM-1 ^30^ were successfully synthesized. Methodologies adopting commercially available organic additives, such as cetyltrimethylammonium (CTA) and amphiphilic organosilane (TPOAC), with the presence of hexamethylenimine (HMI) as the customized OSDA were also developed.^15,26^ The epitaxial growth method developed by Tang and co-workers with the presence of hexagonal boron nitride (h-BN) is also quite effective.^31^

The distribution of T atoms (such as Al and Ti) within the zeolite framework plays a crucial role in determining the catalytic performance.^20,32,33^ Acid sites located at the channel intersections of ZSM-5 promote the cracking of 3-methylpentane via a bulky transition state, but reduce the stability in the cracking of methylcyclohexane due to increased coke formation.^34^ Yokoi et al. demonstrated that the use of Na^+^ and TPA^+^ as SDAs guided the positioning of Al atoms in either the 10-*ring* channels or the channel intersections of ZSM-5.^34^ In MOR and FER zeolites, Al atoms located in the 8-*ring* channels are ideal for the carbonylation of dimethyl ether to methyl acetate, whereas acid sites located in the 12-*ring* or 10-*ring* channels often cause coke formation and rapid deactivation.^35,36^ Similarly, in MWW zeolites, Ti atoms predominantly positioned in the sinusoidal 10-*ring* channels exhibit unique *trans*-selectivity in the epoxidation of *cis*/*trans*-alkenes with hydrogen peroxide.^37^ Therefore, precise control over the distribution and location of T (Al) atoms in zeolites is essential to optimize the performance for specific applications. To achieve this, several strategies have been developed for fine-tuning Al distribution, which can be broadly categorized into “bottom-up” methods (such as the use of specialized or complex OSDAs, combinations of cations and OSDAs or mixed-oxide precursors) and “top-down” approaches (post-treatments).^32,38,39^

The MWW zeolite framework is composed of eight distinct T sites (T_1_–T_8_ sites).^3^ Among these, the T_1_ site is positioned within the 10-*ring* connecting channels with a T_1_-O-T_1_ angle of ∼180°, the T_2_ and T_3_ sites are located on the external surface of the 12-*ring* semi-supercages pointing to 10-*ring* connecting channels, the T_4_ site is located in the framework pockets which is inaccessible to the reactants, the T_5_, T_7_ and T_8_ sites are located in the 10-*ring* sinusoidal channels with T_7_ and T_8_ sites connecting to the bottom of supercages, and the T_6_ sites exist on the inner wall of supercages.^26,40–42^ The most favorable sites for Al substitutions are T_2_, T_3_ and T_8_ with T_2_ and T_3_ bridging −OH groups exhibiting the strongest Brønsted acidity.^40^ Further studies have corroborated these findings, showing that the stability of Al substitution sites follows the order of T_4_ ≈ T_2_ > T_6_ ≈ T_8_ ≈ T_3_ > T_7_ > T_5_ > T_1_, among which T_7_, T_5_ and T_1_ sites are less stable and thus are unlikely to be occupied.^26,41^ MCM-22 zeolite contains a higher concentration of Al atoms at T_7_ sites with more Bro̷nsted acid sites in the sinusoidal channels and exhibits less Al atoms at T_1_–T_3_ sites than MCM-49, and ZnCrO*_*x*_*-MCM-22 with more Al atoms at T_7_ sites was demonstrated to show superior stability and enhanced selectivity to C_5_–C_11_ in the syngas conversion to gasoline reaction.^43^ By introducing a large amount of cetyltrimethylammonium (CTA) as the exfoliating agent, MWW zeolite nanosheets with an increased concentration of T_2_ acid sites and larger external surface area were prepared and showed better catalytic performance in reactions dealing with bulky molecules.^26^ However, because of the special structural feature of MWW zeolite, there also exist T_2_ sites in the interlayer supercages. Thus, the contribution of T_2_ sites to the enhanced activity is still debatable. Chu and co-workers reported the successful synthesis of MWW zeolite with increased T_2_ sites using cyclohexylamine, but only comparable catalytic performance was achieved.^44^

In the present study, a composite synthetic system with the presence of both TMAdaOH and cyclohexylamine as dual OSDAs was employed for the direct syntheses of MWW zeolites with T_2_ Al sites variations. The crystallization of MWW zeolite was comprehensively investigated. Advanced solid-state NMR characterization was carried out to address the correlation between Al sites and organic species. Catalytic performance of the obtained MWW zeolites in cracking of 1,3,5-triisopropylbenzene (TiPB) and alkylation of benzene with cyclohexene was determined and analyzed to illustrate the contribution of T_2_ Al sites.

## 2 Experimental Section

### 2.1 Materials

Sodium aluminate (NaAlO_2_, with Al_2_O_3_ 40–45% and Na_2_O 25–35%), sodium hydroxide (NaOH, 99.5%), ammonium chloride (NH_4_Cl, 99.5%), cyclohexylamine (99.5%), benzene (99.5%) and cyclohexene (99.5%) were bought from Sinopharm chemical reagent Co. Si-Sol (Ludox HS-40, 40%) and 1,3,5-triisopropylbenzene (TiPB, 99%) were supplied by Sigma-Aldrich. N,N,N-Trimethyl-1-adamantylammonium hydroxide (TMAdaOH, 25%) and hexamethylenimine (HMI, ≥98%) were bought from Shanghai Aladdin Biochemical Technology Co. Deionized water was made in the laboratory. All reagents were directly used without further purification.

### 2.2 Synthesis

In a typical synthesis process, a specified amount of NaOH, TMAdaOH and cyclohexylamine was mixed with deionized water and stirred for 1 h. Subsequently, Si-Sol and NaAlO_2_ were added under continuous stirring, and the mixture was aged for 3 h. The result starting gels with molar compositions of 1 SiO_2_:0.033 Al_2_O_3_:0.001–0.05 TMAdaOH:0.15 cyclohexylamine: 0.12 NaOH:18 H_2_O, were transferred into a Teflon-lined autoclave and subjected to hydrothermal crystallization at 160 °C for 3 days under rotation (20 rpm) with a heating rate of 10 °C/min. After crystallization, the products were centrifuged, thoroughly washed and dried overnight. The as-synthesized products, labeled as MWW-*x*-0.15 (where *x* represents the molar ratio of TMAdaOH/SiO_2_ and 0.15 indicates the molar ratio of cyclohexylamine/SiO_2_), were then calcined at 550 °C for 6 h to remove the organics and yield Na-form zeolites. For comparison, conventional MCM-22 zeolite was synthesized following a previously reported method using HMI as the OSDA.^16^ For 10 L scale-up synthesis, the starting gels were hydrothermal crystallization at 160 °C for 3 days under stirring (80 rpm) with a heating rate of 1 °C/min.

To obtain H-form zeolites, Na-form zeolites underwent a triple ion-exchange process using a 0.5 M NH_4_Cl solution at 50 °C for 3 h with continuous stirring and a solid-to-liquid mass ratio of 1:20. The exchanged materials were then dried and calcined at 550 °C for 6 h, resulting in the formation of H-form zeolites.

### 2.3 Characterization

X-ray powder diffraction (XRD) patterns were acquired on a Panalytical X PERPRO X-ray diffractometer (40 kV, 40 mA) using CuKα (λ = 1.5406 Å) radiation in the 2θ range 2–50°. Scanning electron microscopy (SEM) images were captured on a Hitachi S-4800II electron microscope. Transmission electron microscopy (TEM) images were performed on a FEI Tecnai 20 S-TWIN instrument. N_2_ adsorption–desorption isotherms were obtained by using a BELSORP BEL-MAX instrument at −196 °C. ICP-OES spectra were obtained by using an Agilent 725ES instrument. Solid-state ^1^H, ^27^Al and ^29^Si Magic Angle Spinning (MAS) Nuclear Magnetic Resonance (NMR) spectra were acquired on a Varian 400WB NMR spectrometer at 9.4 T. ^13^C MAS, ^1^H–^13^C (or ^27^Al) Cross-Polarization (CP) and 2D ^1^H–^13^C (or ^27^Al) heternuclear correlation (HETCOR) NMR spectra were obtained from a Bruker-Avance III 500 spectrometer at 11.7 T. The ^1^H–^13^C (or ^27^Al) CP experiments were performed on a 4 (or 7) mm probe with a spinning rate of 12 (or 5) kHz using hexamethylbenzene (or kaolinite) as the reference sample to obtain the Hartmann–Hahn conditions. For 2D ^1^H–^13^C (or ^27^Al) HETCOR NMR, an increment of the mixing time by 16 (or 20) μs was employed for each data point in the ^1^H indirect dimension and 360 (or 480) scans were used in the ^13^C (or ^27^Al) dimension.^45^ 2D ^27^Al multiple-quantum magic angle spinning (MQ MAS) NMR spectra were acquired at 18.8 T on a Bruker Avance III 800 spectrometer equipped with a 3.2 mm probe using a pulse width of 5.3 μs for the first high-power excitation and a pulse width of 1.7 μs for the second high-power transition with a high-power RF field strength of 150 kHz; the “soft” π/2 pulses of low-power were 12.0 μs.^46^ The ^27^Al MAS NMR spectra were acquired using a small-flip angle technique with pulse lengths of 0.50 and 0.33 μs (<π/12) and a recycle delay of 1 s at 9.4 and 18.8 T, respectively. The ^27^Al MAS spectra at 18.8 T were fitted using a quadrupolar line shape with a CZ simple model in the DMFIT software.^47^ NH_3_-TPD curves were measured using an Altamira AMI-3300 instrument. IR spectra were collected on a Nicolet Model 710 spectrometer with the adsorption of pyridine (Py-IR) and 2,6-di-tert-butyl-pyridine (2,6-DTBPy-IR) at 150 °C. In the Py-IR spectra, integral areas of the adsorption bonds at ∼1545 and ∼1453 cm^–1^ were used to determine the concentrations of Brønsted acid (B acid) and Lewis acid (L acid) sites by using extinction coefficients (ε_B_ = 1.67 cm/μmol and ε_L_ = 2.22 cm/μmol), respectively;^48^ and in the 2,6-DTBPy-IR spectra, the integral area of the bonds at ∼1616 cm^–1^ was employed to calculate the amount of external Brønsted acid (B_Ext_ acid) sites (ε_BExt_ = 1.67 cm/μmol).^49^ Thermal analysis, including thermogravimetry (TG) and derivative thermogravity (DTG) analysis, was performed on a SDT Q600 V20.9 Build 20 thermal analyzer instrument using air as the purge gas from 50–900 °C with a heating rate of 10 °C/min. Benzene diffusion was evaluated using a computer-controlled intelligent gravimetric analyzer (IGA) from Hiden Analytical Ltd. (Warrington, UK), following the experimental protocols described in earlier reports.^50,51^

### 2.4 Catalytic Performance Tests

The catalytic cracking of TiPB was performed in a fixed-bed pulse microreactor equipped with an Agilent automatic liquid sampler (ALS).^52^ In a typical experiment, 50 mg of H-form catalyst (40–60 meshes) was mixed with 200 mg of quartz sand and loaded into a reactor with an inner diameter of 4 mm. Prior to the reaction, the catalyst was pretreated at 350 °C for 0.5 h, followed by cooling to 300 °C. Subsequently, 1 μL of TiPB was injected via the ALS for a total of 30 pulses. The products from each pulse were analyzed using an Agilent 8890 gas chromatograph (GC) equipped with an INNOWAX column. The conversions of TiPB and the selectivity for benzene were calculated using the following formulas:

$$\mathrm{Conversion}\%=\left(1-\frac{c_{TiPB}}{\sum c_{i}}\right)\times 100\%$$ 1

$$\mathrm{Selectivity}\%=\frac{c_{Benzene}}{\sum c_{i}-c_{TiPB}}\times 100\%$$ 2

in which C*_i_* represents the molar concentration of product *i* with a benzene ring.

Liqud-phase alkylation of benzene with cyclohexene was carried out in a continuous fixed-bed reactor with detailed experimental conditions previously described elsewhere.^25^ The reaction was performed at 150 °C under 2.0 MPa with a benzene-to-cyclohexene molar ratio of 10 and total weight hourly space velocity (WHSV) of 3.65 h^–1^.

## 3 Results and Discussion

### 3.1 Physiochemical Properties of the MWW Zeolites

The FER phase was exclusively observed in the product (FER-0–0.15) when no TMAdaOH was introduced (Figure 1), since cyclohexylamine could be used as the OSDA for the synthesis of FER-type zeolite.^44^ In the composite synthetic system with both cyclohexylamine and TMAdaOH, even a trace amount of TMAdaOH (*x* = 0.001), pure MWW phases was obtained at 160 °C (Figure 1). It is worth noting that, with the increase of TMAdaOH (*x* = 0.001–0.05), the diffraction peaks corresponding to the (001) and (002) faces gradually emerge and increase to closely resemble those of conventional as-synthesized MCM-22 zeolite (MCM-22 zeolite precursor, MCM-22­(P)) using HMI as the OSDA, reflecting the evolution of the structure from a three-dimentional (3D) fully connected MWW framework toward 2D layered MCM-22­(P). The reason behind this is that TMAda^+^ stabilizes the 12-*ring* cage, while cyclohexylamine stabilizes the smaller sinusoidal 10-*ring* channels.^11^ Upon calcination, all samples turned into 3D fully connected frameworks without any detectable FER phase (Figure S1).

**Figure 1:**
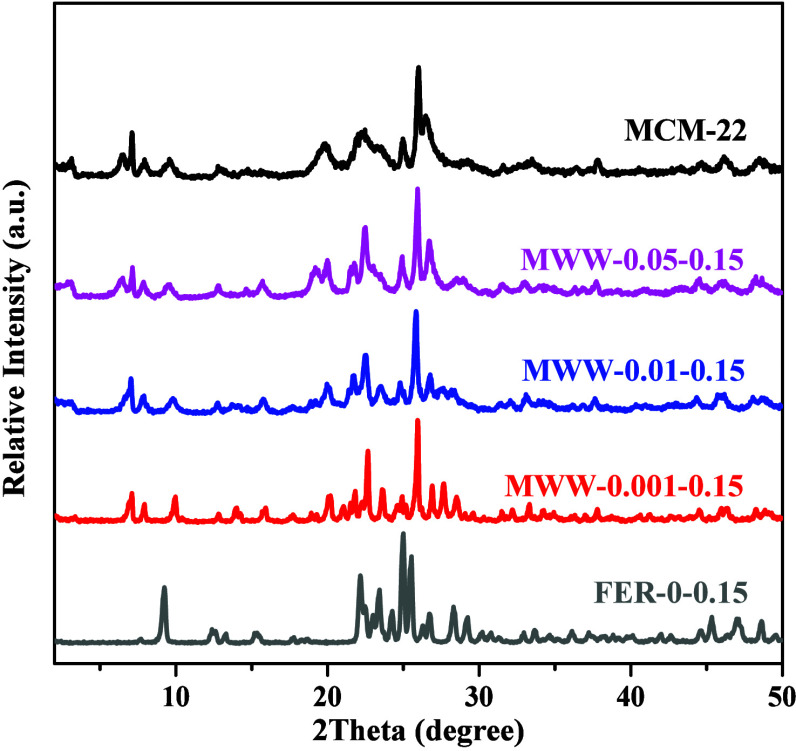
XRD patterns of zeolites synthesized with varying amounts of TMAdaOH and conventional MCM-22.

To further investigate the properties of these zeolites, SEM and N_2_ adsorption–desorption measurements were conducted, as shown in Figures S2–S4. The results reveal that all MWW type zeolites possess nanosheet morphology. Additionally, the crystal sizes of the MWW-*x*-0.15 series zeolites are in the range of 300–400 nm, which are smaller than those of conventional MCM-22 zeolites (Figure S2). The FER zeolite exhibits a small block-like morphology with a thickness of ∼50 nm, as illustrated in Figure S3. Both FER-0–0.15 and MWW-0.001–0.15 zeolites display type I adsorption isotherms, indicative of microporous characteristics. In contrast, MWW-0.01–0.15, MWW-0.05–0.15 and MCM-22 exhibit a combination of type I and type IV adsorption isotherms (Figure S4).^26,52^ The textural properties of the MWW zeolite samples are summarized in Table 1. Both the external surface area (S_Ext_) and total volume (V_Total_) increase with the increase of TMAdaOH content in the starting gel, ranging from 32 to 153 m^2^/g and 0.54 to 0.79 cm^3^/g, respectively. Notably, the S_Ext_ of MWW-0.01–0.15 (114 m^2^/g) surpasses that of MCM-22 (102 m^2^/g). In contrast, the micropore volume (V_Micro_) decreases from 0.21 to 0.15 cm^3^/g, reflecting a well-crystallized structure across the samples.^15^ Additionally, TEM images (Figure 2) reveal that the thickness decreases progressively with the increase of TMAdaOH content. MWW-0.05–0.15 exhibits a minimum thickness of ∼10 nm (corresponding to ∼4 layers), compared to MCM-22 with a thickness of ∼15 nm (∼6 layers). These observations suggest that TMAdaOH serves two roles, functioning as an OSDA and an interlayer growth inhibitor,^53^ effectively modulating the layered structure.

**Figure 2:**
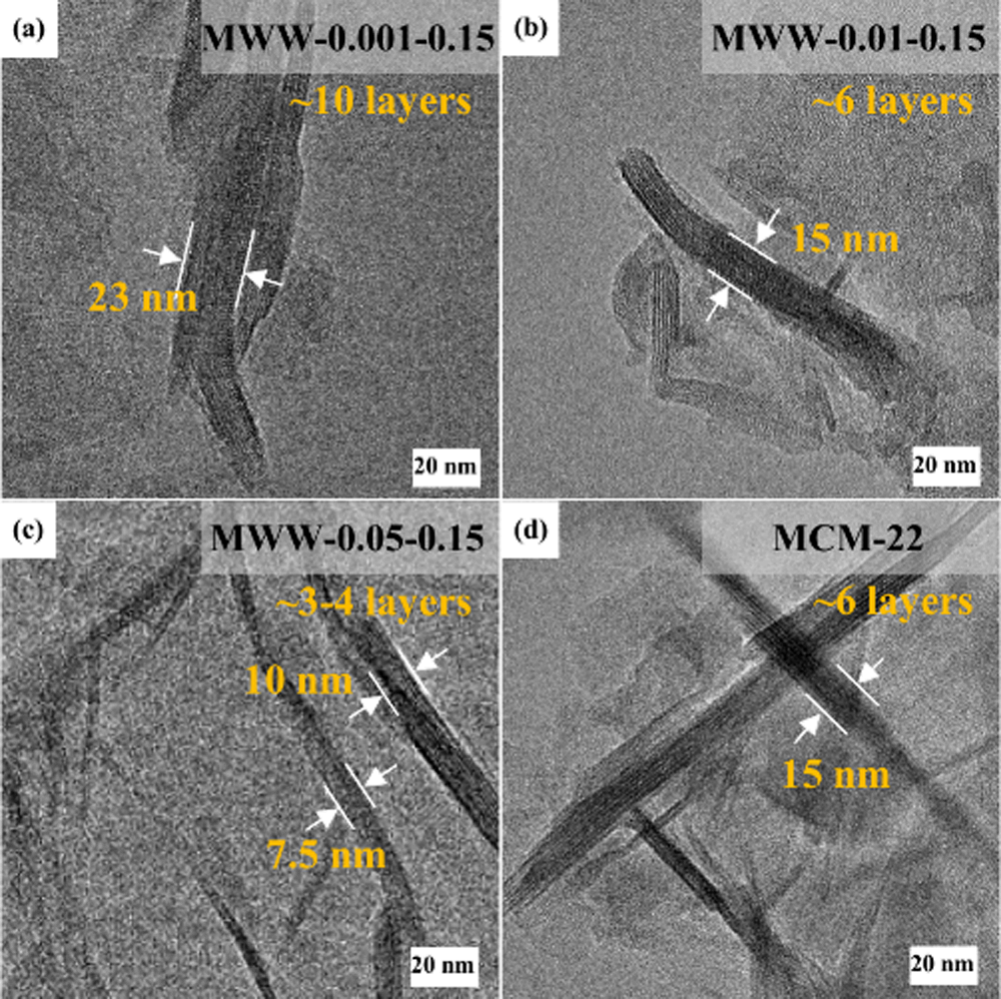
TEM images of (a–c) MWW zeolites produced with varying amounts of TMAdaOH and (d) conventional MCM-22.

**Table 1:** Textural Properties of MWW Zeolites Produced with Varying Amounts of TMAdaOH and Conventional MCM-22

Sample	S_BET_ (m^2^/g)^a^	S_Ext_ (m^2^/g)^b^	V_Total_ (cm^3^/g)^c^	V_Micro_ (cm^3^/g)^b^
MWW-0.001–0.15	571	32	0.54	0.21
MWW-0.01–0.15	522	114	0.72	0.17
MWW-0.05–0.15	459	153	0.79	0.15
MCM-22	510	102	0.74	0.17

a BET method.

b t-Plot method.

c N_2_ volume adsorbed at P/P_0_ = 0.99.

The crystallization of MWW zeolites requires precise control of both synthesis temperature and time. As shown in Figure S5, only an amorphous solid was obtained when the gel was heated at 150 °C. Higher temperature (160 °C) resulted in the crystallization of pure MWW phase. However, at 170 °C, a mixed MWW/FER phase was observed. The effects of synthesis time on zeolite crystallization were also studied. As shown in Figure S6, when the synthesis time was less than 12 h, only the amorphous phase was observed. The intensities of diffraction peaks increased with the increase of synthesis time, and the peaks corresponding to MWW phase started to emerge after 16 h. Fully crystallized MWW zeolites were obtained within 1–4 days, as indicated in Figures S6, S7 and Table S1. However, when the crystallization was extended beyond 5 days, the FER phase began to emerge and eventually became the dominant product. The above results highlight the crucial role of synthesis temperature and time in achieving highly crystalline MWW zeolites while suppressing the formation of competing phases such as FER.

The influence of cyclohexylamine on the crystallization process is illustrated in Figures S8–S10. Without the addition of cyclohexylamine, only the amorphous phase was obtained, indicating the importance of cyclohexylamine in facilitating the crystallization of the MWW phase. Fully crystallized MWW zeolites were obtained with a suitable amount of cyclohexylamine in the range of 0.15–0.30, and the products exhibited comparable textural properties, as summarized in Table S1. As shown in Figure S11, the effect of Na^+^ was also investigated. CHA-type zeolite was obtained at a lower Na^+^/SiO_2_ molar ratio of 0.09; in contrast, pure phase MWW zeolites were observed when the Na^+^/SiO_2_ molar ratio was maintained within the range of 0.12–0.15. However, a mixed MWW/FER phase was obtained at high Na^+^/SiO_2_ molar ratio (0.18). These findings emphasize that the presence of an appropriate amount of Na^+^ (0.12–0.15) is crucial for the crystallization of MWW zeolites. Figure 3 provides an overview of the TMAdaOH-cyclohexylamine composite syntheisis system, demonstrating the synergy between TMAdaOH and cyclohexylamine in the starting gels is essential for directing the formation of the MWW structure, and TMAdaOH possesses a more significant impact on the textural properties of the MWW zeolites compared to cyclohexylamine. Under optimized conditions, pure-phase MWW zeolites were successfully synthesized at a larger scale, achieving a yield of over 80% in a 10 L autoclave. Importantly, as shown in Figures S12–S14, the MWW-0.01–0.15 and MWW-0.05–0.15 samples produced on this scale are well-crystallized and exhibit characteristics consistent with those obtained from synthesis in a smaller 100 mL autoclave. The increase in V_Macro_ for MWW-0.05–0.15–10L compared to MWW-0.05–0.15 could be attributed to the differences in crystallization conditions, including stirring speed, heating rate and the role of TMAda^+^ at higher concentrations. This demonstrates the scalability and reproducibility of the synthesis process under controlled conditions.

**Figure 3:**
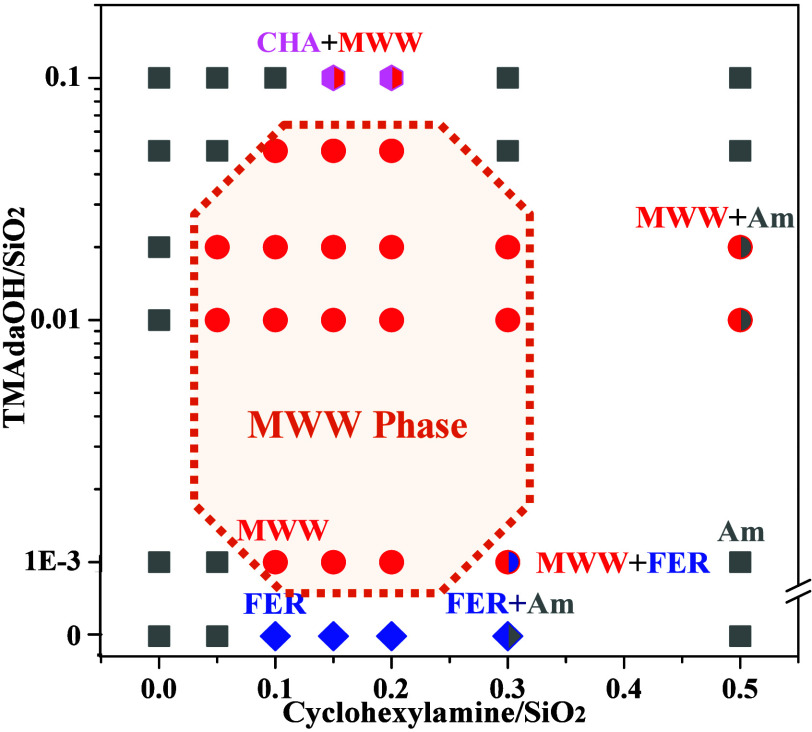
Overall landscape of the crystallization products in the TMAdaOH-cyclohexylamine composite synthesis system.

### 3.2 Role of TMAdaOH and Cyclohexylamine as the OSDAs

In order to ascertain the roles of TMAdaOH and cyclohexylamine in the synthesis of MWW zeolites, solid-state ^13^C MAS and ^1^H–^13^C CP MAS NMR spectra of the as-synthesized MWW zeolites were examined, as shown in Figures 4(a) and S15, respectively. Basing on the ^13^C chemical shifts of the two OSDAs in liquid ^13^C NMR, the solid-state ^13^C NMR signals are assigned as follows:^11,44^ the peak at 24.9 ppm corresponds to the overlapping C(1’) and C(2’) resonances of cyclohexylamine; 30.7 ppm is attributed to the overlapping C(1) and C(2) resonances of TMAda^+^; 35.5 ppm represents the overlapping C(3) and C(3′) resonances of TMAda^+^ and cyclohexylamine, respectively; 48.0 ppm is assigned to the C(4) resonance of TMAda^+^; 49.8 ppm corresponds to the C(4’) resonance of cyclohexylamine; and 73.3 ppm represents the C(5) resonance of TMAda^+^. The relative NMR peak intensities of the two OSDAs in the as-synthesized MWW zeolites varies significantly with the variations of TMAdaOH amount adopted in the starting gels. Taking the C(1) and C(2) peak of TMAdaOH and the C(1’) and C(2’) peak of cyclohexylamine for example, as the TMAdaOH content increases, the intensities of the C(1) and C(2) resonances associated with TMAdaOH show a marked rise, while the intensities of the C(1’) and C(2’) resonances attributed to cyclohexylamine decrease significantly. Using the above two shifts as quantitative references, as the TMAda^+^/cyclohexylamine molar ratio increases from 0.001:0.15 to 0.05:0.15 during sample preparation, the actual molar fraction in the as-synthesized MWW-*x*-0.15 samples shifts significantly from 17:83 to 71:29, indicating the concentration of cyclohexylamine decreases significantly upon the addition of TMAdaOH. This finding is likely due to that the presence of TMAda^+^ affected the distribution and interaction of cyclohexylamine within the framework. Solid-state ^1^H MAS NMR spectra of the as-synthesized MWW-*x*-0.15 zeolites provided further information for the role of the dual-OSDA in zeolites crystallization. As presented in Figure 4(b), the chemical shifts at 1.0 to 1.5 ppm can be assigned to overlapping ^1^H moieties from cyclohexylamine (H(1’), (H(2’)), while shifts at 1.5 to 2.0 ppm are attributed to TMAda^+^ (H(3)), cyclohexylamine (H(3′), (H(5′)). Signals at 2.5 to 3.0 ppm arise from TMAda^+^ (H(1), (H(2)), cyclohexylamine (H(1’), (H(2’)), (H(4’)). Notably, a distinct signal at ∼7 ppm, attributed to quaternized cyclohexylamine (H(5′)), is only observed in MWW-0.001–0.15, indicating a high degree of cyclohexylamine quaternization in this sample. Increasing the concentration of TMAdaOH results in the disappearance of the signal at ∼7 ppm on the MWW-0.01–0.15 and MWW-0.05–0.15 samples. These findings demonstrate that the concentration of TMAda^+^ plays a critical role in determining the extent of cyclohexylamine quaternization and the modes of dual OSDA incorporation into the zeolite framework; and these results might explain the different connection modes observed in the MWW zeolites. Similar to the behavior of HMI as an OSDA in the synthesis of MCM-22 zeolites (Figure S16), both TMAdaOH and cyclohexylamine actively participate in the formation of the MWW structure, functioning as dual OSDAs and playing a synergistic role in structure-directing.

**Figure 4:**
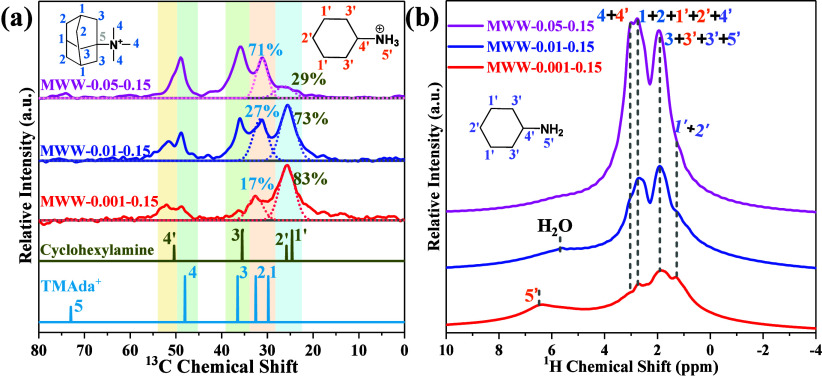
(a) Solid-state ^13^C NMR and (b) ^1^H NMR spectra of MWW zeolites synthesized with varying amounts of TMAdaOH.

The thermogravimetric (TG) and differential thermogravimetric (DTG) profiles of MWW-*x*-0.15 zeolites and conventional MCM-22 are presented in Figure S17. Weight loss of the as-synthesized MWW zeolite can be divided into three stages: (A) < 180 °C is attributed to the desorption of physically adsorbed H_2_O; (B) 180–480 °C is assigned to the removal of organics (TMAda^+^, cyclohexylamine or HMI) located within the surface semi-supercages and interlayer supercages; (C) > 480 °C is due to the decomposition of organics in the 10-*ring* sinusoidal channels.^44,54^ The total weight loss of the MWW-*x*-0.15 zeolites increases progressively with higher TMAdaOH content, reaching 15.6%, 16.2%, and 20.1%, respectively (Table S2). Notably, weight loss in stage C exhibits a slight increase (5.4%–6.6%) with higher TMAdaOH, while a significant rise is observed in stage B, ranging from 6.4% to 11.6%, with the majority of weight loss in MWW-0.05–0.15 occurring in this stage. The DTG curves (Figure S17­(b)) reveal distinct thermal decomposition behaviors. All MWW-*x*-0.15 zeolites display two primary peaks near 318 °C and between 605–631 °C, corresponding to organic species occluded in 12-*ring* and 10-*ring* channels, respectively. MWW-0.05–0.15 exhibits an additional pronounced peak at approximately 252 °C attributed to the removal of TMAda^+^ located in the surface semi-supercages, where it is more readily eliminated from the framework. For comparison, MCM-22 exhibits decomposition peaks at higher temperatures of ∼424 °C (stage B) and 638 °C (stage C), consistent with the stronger interaction of HMI (used as the OSDA) with the zeolite framework. These findings suggest that TMAda^+^ predominantly resides in the interlayer supercages and surface semi-supercages, while cyclohexylamine distributed mainly in sinusoidal channels. Furthermore, the interaction of HMI with the MCM-22 framework is notably stronger than that of cyclohexylamine, as reflected by the higher decomposition temperatures in MCM-22. This highlights the differing roles and framework interactions of OSDAs in regulating properties of the obtained zeolites, for instance, the distribution of framework aluminum atoms.

Figure S18 displays the low magnetic field (9.4 T) ^27^Al MAS NMR spectra of as-synthesized, freshly calcined, and calcined (∼1 year ago) MWW-0.01–0.15 samples. The extra-framework aluminum signal (∼0 ppm) remains consistent, with fractions of 10.2% and 10.4%, demonstrating a long-term framework stability of the MWW zeolites. According to the previous reports, the MWW framework comprises eight distinct T-sites (Scheme 1), and the tetrahedral aluminum sites are categorized into three groups based on the ^27^Al MAS NMR chemical shifts:^26^ (I) a signal at ∼50 ppm corresponding to the overlap of T_6_ and T_7_; (II) a signal at ∼56 ppm assigned to the overlap of T_1_, T_3_, T_4_, T_5_ and T_8_; and (III) a signal at ∼61 ppm attributed to T_2_. High magnetic field (18.8 T) solid-state ^27^Al MAS NMR and 2D ^27^Al Multiple-Quantum Magic-Angle Spinning (MQMAS) NMR were performed to further investigate the influence of TMAdaOH on the spatial distribution of aluminum sites within the framework (Figure 5). The ^27^Al MAS NMR spectra of the MWW zeolites were deconvoluted by three groups using their isotropic chemical shifts and PQ parameters obtained from the 2D MQMAS NMR spectra, and the integrated area proportions of each group are summarized in Table 2. For comparison, the distribution of aluminum sites in conventional MCM-22 zeolite with similar Si/Al ratio synthesized using HMI as the OSDA was also investigated. Notably, all MWW-*x*-0.15 samples exhibit a higher fraction of T_2_ aluminum sites (>24%) compared to conventional MCM-22 zeolite (21.8%). According to pervious report,^44^ the T_2_ site aluminum-enriched nature of MWW-*x*-0.15 samples is probably attributed to the balance between Na^+^ ions and quaternized cyclohexylamine affecting aluminum distribution in the framework. Herein, as the TMAda^+^/cyclohexylamine ratio in the starting gel increases, the proportion of T_2_ sites gradually decreases from 28.3% to 24.2%. This trend suggests again that TMAda^+^, predominantly located in 12-*ring* channels (interlayer supercages and the surface semi-supercages), significantly affects the actual TMAda^+^/cyclohexylamine ratio in the as-synthesized MWW zeolites and preferentially alters T-site occupancy, resulting in variations in aluminum distribution within the framework. These findings underscore the role of dual OSDAs in modulating the structural and chemical environment of the aluminum sites in MWW zeolites.

**Figure 5:**
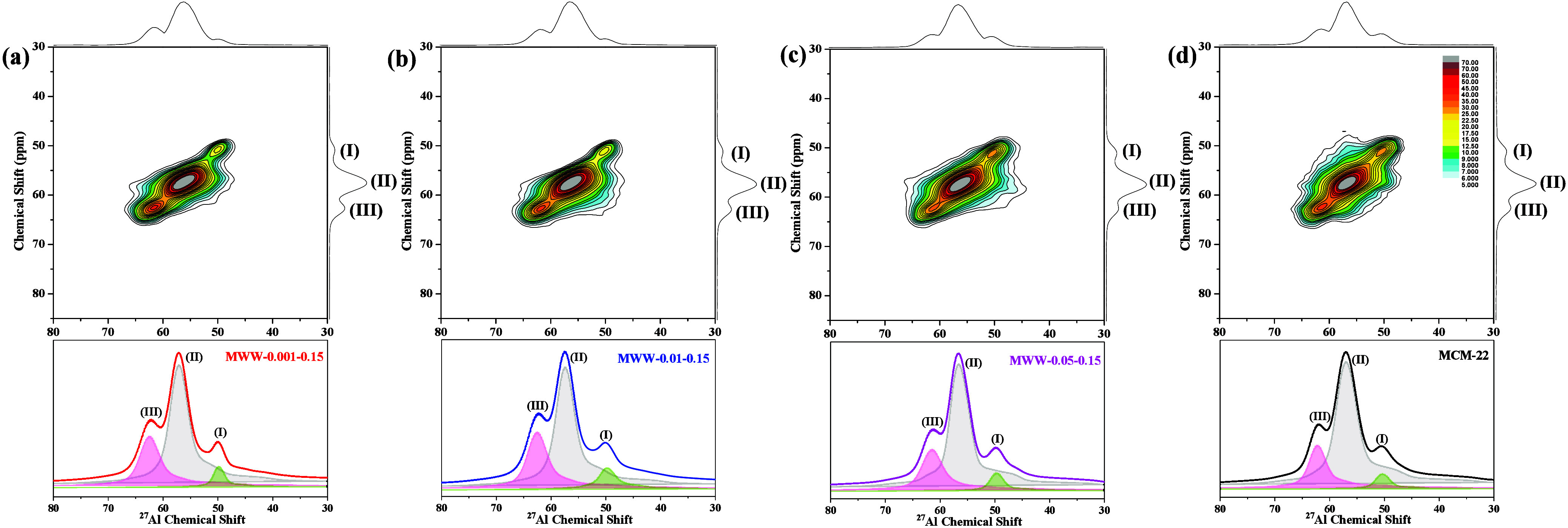
2D ^27^Al MQ MAS NMR spectra of (a–c) MWW zeolites produced with varying amounts of TMAdaOH and (d) conventional MCM-22. Experimental and simulated ^27^Al MAS NMR spectra at the bottom show the proportion of Al sites in different groups.

**Table 2:** Isotopic Chemical Shift (δ_iso_) and Quadruple Interaction Parameter (P_Q_) of Different Group Al Sites and Their Relative Intensities on Different Zeolites Obtained from Simulations of the ^27^Al MAS NMR Spectra

			Proportion (%)			
Al sites	δ_iso_ (ppm)	P_Q_ (MHz)	MWW-0.001–0.15	MWW-0.01–0.15	MWW-0.05–0.15	MCM-22
Group (I)	50.7	2.44	7.8	10.9	8.1	7.4
Group (II)	57.3	2.24	63.9	61.5	67.7	70.8
Group (III)	62.3	2.34	28.3	27.6	24.2	21.8

Solid-state NMR HETCOR is a powerful spectroscopic technique that can be used in the investigation of coupling interactions between various nuclei within a material to show detailed information about their spatial proximity.^2^ To investigate the interaction between OSDAs and aluminum sites within the zeolite framework, solid-state 2D ^1^H- ^27^Al and ^1^H–^13^C HETCOR NMR studies were conducted on the as-synthesized MWW-0.001–0.15 and MWW-0.05–0.15 samples (Figure 6).^55^ The combined analysis of ^1^H–^27^Al and ^1^H–^13^C HETCOR NMR spectra of MWW-0.001–0.15 (Figure 6(a) and (b)) provide a comprehensive understanding of the role of the OSDAs. For the as-synthesized MWW-0.001–0.15 zeolites with a lower TMAda^+^/cyclohexylamine ratio, TMAda^+^ (C(1)–C(3)) and quaternized cyclohexylamine (C(1’)–C(3′)) correlate with the group (I) and (II) ^27^Al sites, while TMAda^+^ (C(4)) and cyclohexylamine (C(4’)) are probably linked to the group (III) ^27^Al sites. The as-synthesized MWW-0.05–0.15 zeolite with a higher TMAda^+^/cyclohexylamine ratio shows obvious correlation between TMAda^+^ (C(1)–C(3)) and group (I) to (III) ^27^Al sites, whereas the correlation between TMAda^+^ (C(4)), cyclohexylamine (C(4’)) and group (III) ^27^Al sites is unobservable. The existence of cyclohexylamine in the as-synthesized MWW-0.05–0.15 zeolite is confirmed by ^13^C MAS and ^1^H–^13^C CP NMR spectra (Figures 4(a) and S15). These observations indicate that the TMAda^+^/cyclohexylamine ratio plays important role in directing different distributions of the framework Al atoms, lower TMAda^+^/cyclohexylamine ratio, more Al atoms in group (III), consistent with the results in ^27^Al MAS and MQMAS experiment (Figure 5). Considering the TGA result that a characteristic peak (∼252 °C) attributed to TMAda^+^ located in the surface semi-supercages is obviously observed when increasing the TMAda^+^/cyclohexylamine ratio, it suggests the effect of TMAdaOH in modulation of the proportion of external surface Al atoms.

**Figure 6:**
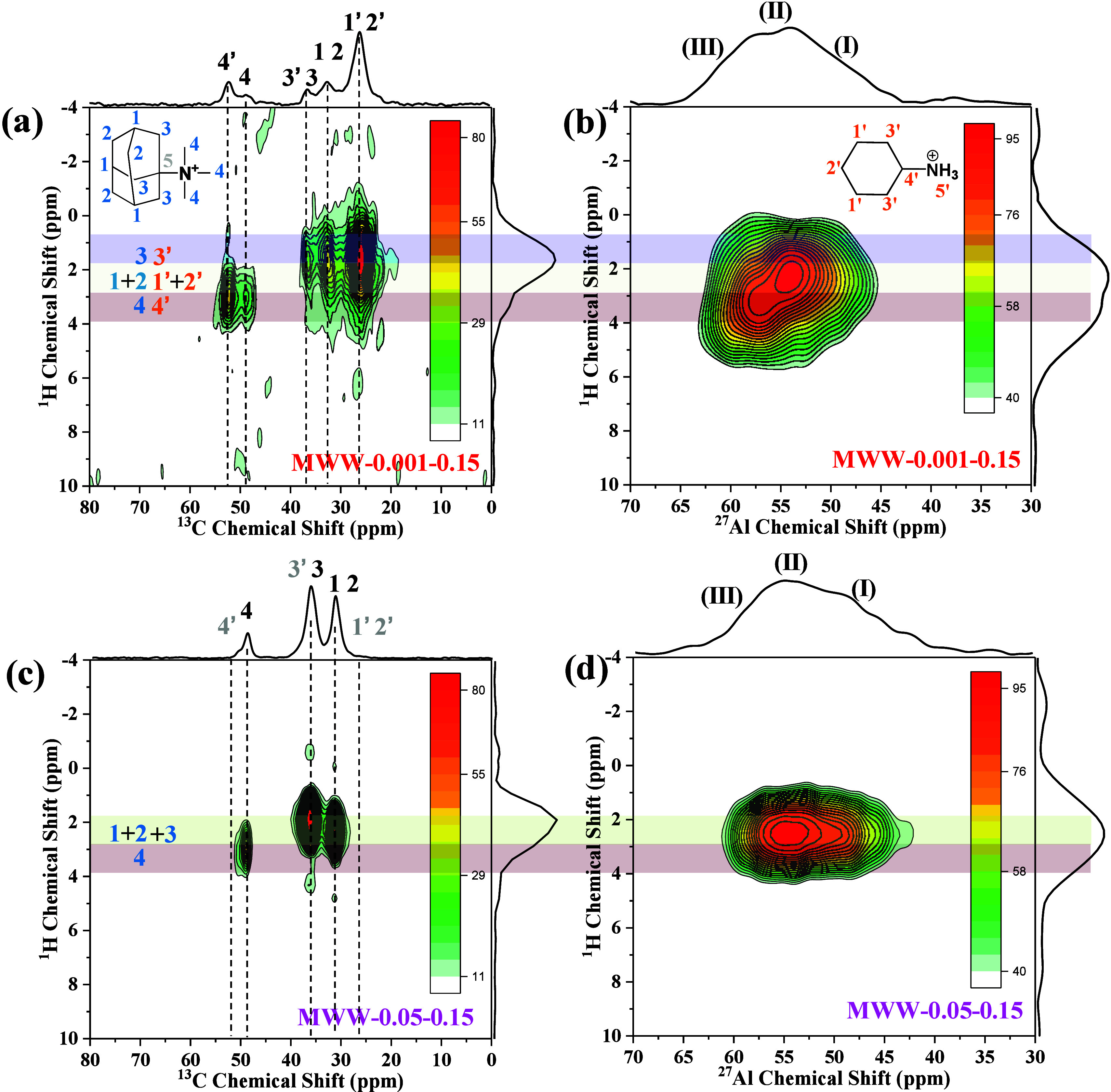
2D ^1^H–^13^C (a and c) and ^1^H–^27^Al (b and d) HETCOR NMR spectra of MWW-0.001–0.15 and MWW-0.05–0.15.

MWW zeolites synthesized with different amounts of TMAdaOH exhibit comparable Si/Al ratios. The acid sites of the MWW zeolites were characterized using a combination of NH_3_-TPD, FTIR and NMR techniques, with the corresponding data presented in Figures S19–S22 and summarized in Tables 3 and S3. According to the NH_3_-TPD results (Figure S19), the acid content of H-form MWW-*x*-0.15 zeolites decreases as the TMAdaOH content increases. The increase of TMAda^+^ also causes the slight increase of the Si/Al ratio (Table 3). One possible reason is that the charge density of TMAda^+^ is smaller than those of Na^+^ and quaternized cyclohexylamine.^32^ Among the samples, MWW-0.001–0.15 exhibits the highest total acid amount at 1160 μmol/g, along with a strong acid strength characterized by a peak centered at 374 °C. In contrast, other MWW zeolites show reduced acid content, ranging from 949–887 μmol/g, and a slightly lower strong acid peak centered at ∼345 °C. Furthermore, MWW-0.01–0.15 and MWW-0.05–0.15 display weaker acidity, with properties comparable to those of conventional MCM-22.

**Table 3:** Physicochemical Properties of MWW Zeolites Produced with Varying Amounts of TMAdaOH and Conventional MCM-22

Sample	Si/Al^a^	Acid Amount (μmol/g)^b^	B_Ext/150 °C_ (μmol/g)^c^	Strong Acid (%)^b^	D_eff_/L^2^ ^d^	T_2(Ext)_ (%)^e^
MWW-0.001–0.15	14.4	1160	38	44.3	4.5 × 10^–4^	0.93
MWW-0.01–0.15	14.7	949	72	36.4	9.7 × 10^–4^	2.09
MWW-0.05–0.15	15.3	873	83	33.8	11.4 × 10^–4^	2.30
MCM-22	15.1	887	49	34.1	7.3 × 10^–4^	1.20

a Measured by ICP-OES.

b Calculated based on NH_3_-TPD curves.

c Determined by 2,6-DTBPy-IR spectra (external B acid sites at 1616 cm^–1^).

d Calculated based on Figure 8.

e Calculated based on B_Ext_/Acid Amount × T_2_.

The Py-IR spectra (Figure S20) reveal absorbance peaks at ∼1545 and ∼1453 cm^–1^, corresponding to Brønsted and Lewis acid sites, respectively.^48,49^ All the samples exhibit comparable amounts of Brønsted and Lewis acid sites (Table S3). In the −OH IR spectra, the peak at ∼3740 cm^–1^ is attributed to terminal Si–OH groups located on the external surface of the zeolites, while the peak at ∼3620 cm^–1^ corresponds to the stretching vibration of bridging Si–OH groups (Si–OH-Al) within the zeolite framework.^52^ As depicted in Figure S21, MWW-0.01–0.15 and MWW-0.05–0.15 exhibit similar terminal Si–OH groups and a higher proportion of bridging Si–OH groups compared to conventional MCM-22, with MWW-0.001–0.15 showing the strongest bridging Si–OH peak, consistent with the NH_3_-TPD findings. Additionally, the ^29^Si MAS NMR was employed to analyze the chemical environment of silicon within the samples. The peak at approximately −93 ppm is assigned to terminal defect Si–OH sites (Q_2_, Si­(OSi)_2_(OH)_2_) within the MWW framework.^25,31^ As shown in Figure S22, MWW-0.001–0.15 and MWW-0.01–0.15 exhibit lower Q_2_ peak intensities, indicating a more intact framework. In contrast, MWW-0.05–0.15 displays a Q_2_ peak intensity approaching that of conventional MCM-22, suggesting a slightly less integrated framework. Meanwhile, the stability and framework aluminum environment of MWW-0.01–0.15 were examined using ^27^Al NMR. These findings highlight the robust structural integrity and acid site distribution of the MWW zeolites, which are influenced by the synthesis conditions and composition.

Using 2,6-ditertbutyl-pyridine (2,6-DTBPy) as the probe molecule, 2,6-DTBPy-IR spectroscopy was employed to specifically evaluate the Brønsted acidity located in the external surface semi-supercages.^56^ As shown in Figure 7, the absorbance peak at ∼3370 cm^–1^ corresponds to the N–H stretching vibration of DTBPyH^+^, while the peak at ∼1616 cm^–1^ is attributed to the associated ring vibration mode. The integrated area of this peak was used to quantify the external Brønsted acid content (Table 3). Notably, MWW-0.01–0.15 and MWW-0.05–0.15 display significantly stronger characteristic peaks compared to conventional MCM-22, indicating approximately double the external Brønsted acid amount and correspondingly double the external T_2_ Al content. These findings highlight the enhanced external acidity of the MWW zeolites synthesized with higher TMAdaOH, which may play a critical role in catalytic processes occurring on the zeolite surface.

**Figure 7:**
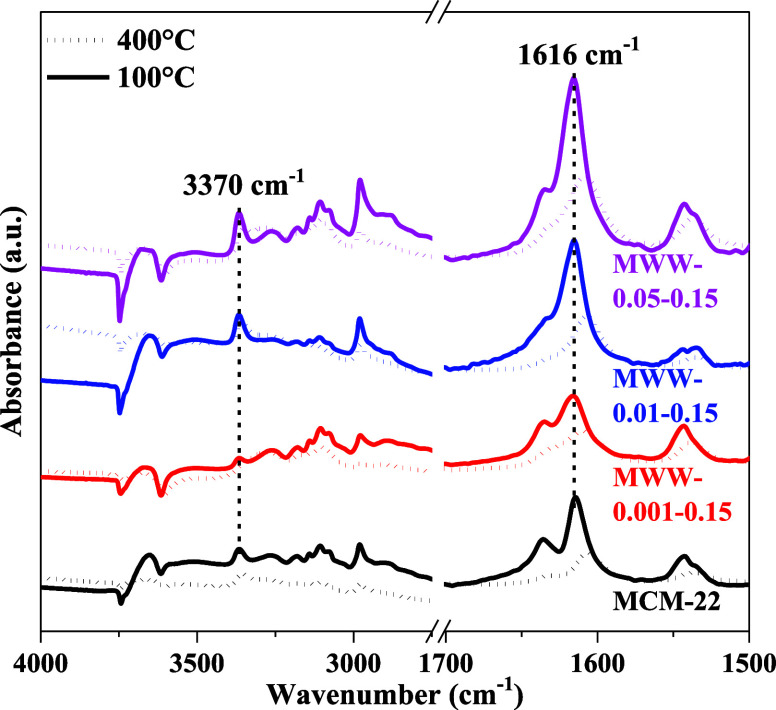
2,6-DTBPy IR spectra of MWW zeolites produced with varying amounts of TMAdaOH and conventional MCM-22.

Benzene was selected as a probe molecule to evaluate the diffusion properties of MWW zeolites using the intelligent gravimetric analyzer (IGA) method.^50,51^ The q­(t)/q(∞) and t^0.5^ plots in Figure 8 exhibit excellent linearity, with the slope of the curves representing the diffusion rate of the zeolites. As summarized in Table 3, the diffusion rates of the samples follow the order: MWW-0.05–0.15 ≈ MWW-0.01–0.15 > MCM-22 > MWW-0.001–0.15. MWW-0.05–0.15 and MWW-0.01–0.15 facilitate faster benzene transport, indicating the critical influence of structural modifications on the diffusion behavior of MWW zeolites.

**Figure 8:**
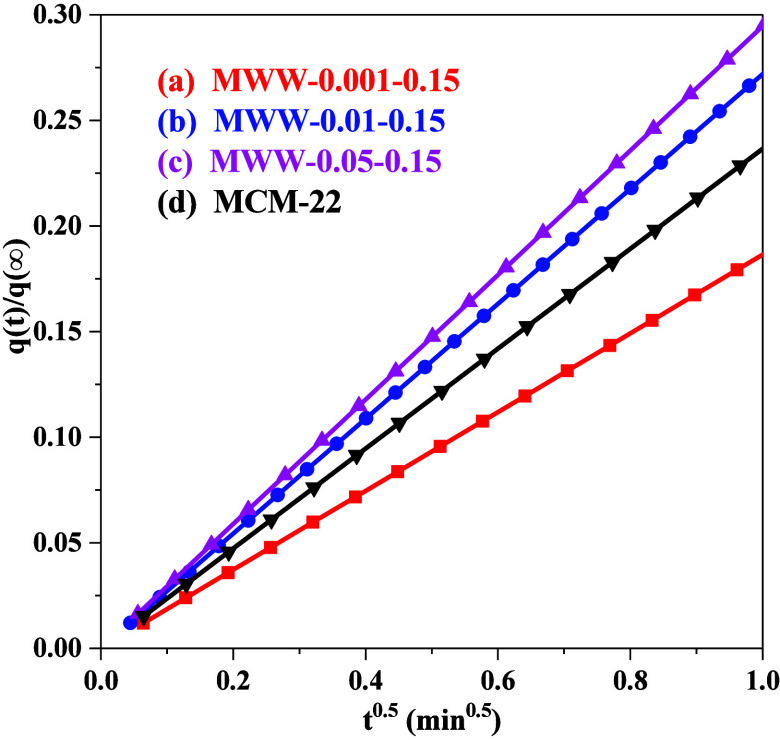
Adsorption rates of benzene of MWW zeolites produced with varying amounts of TMAdaOH and conventional MCM-22.

### 3.3 Catalytic Performance of MWW Zeolites

1,3,5-Triisopropylbenzene (TiPB), with a molecular diameter of 0.94–0.95 nm, was employed as a model reactant to investigate the catalytic contribution of external active sites.^57^ The primary aromatic products of TiPB cracking include diisopropylbenzene (DiPB) isomers, isopropylbenzene (iPB) and benzene. Among these, the selectivity to benzene serves as an indicator of the extent of the cracking reaction and the efficiency of the external active sites.^52^ Figure S23 illustrates the changes in TiPB conversion and benzene selectivity over consecutive pulses on the H-form MWW catalysts. With an increasing number of pulses, MWW-0.001–0.15, with the fewest external active T_2_ sites, exhibits a fastest deactivation rate and lowest benzene selectivity compared to the other samples. In contrast, MWW-0.01–0.15 and MWW-0.05–0.15 demonstrate higher TiPB conversions and longer stabilities than conventional MCM-22. These findings strongly suggest that MWW zeolites synthesized within the TMAdaOH-cyclohexylamine system possess a greater density of external active T_2_ sites, which not only enhances catalytic activity in TiPB cracking but also improves the overall degree of cracking. This highlights the significant role of external active sites in determining both the stability and efficiency in the cracking process.

Cyclohexylbenzene (CHB) is a value-added chemical that serves as a crucial raw material, particularly as an electrolyte additive in lithium-ion battery applications.^25^ As shown in Figure 9, the liquid-phase alkylation of benzene with cyclohexene was evaluated over the MWW catalysts at 150 °C. The conventional MCM-22 catalyst exhibited a lower cyclohexene conversion of 92.5% compared to MWW-0.01–0.15 (99.1%) and MWW-0.05–0.15 (99.3%), while MWW-0.001–0.15 demonstrated the least activity (65.4%) (Figure 9(a) and (b)). Meanwhile, the catalytic activity of MWW zeolite is positively correlated with the aluminum content at external active T_2_ sites (Figure 9(b)), showing little association with the total T_2_ aluminum content (Figure 9(a)). Compared to conventional MCM-22, MWW-0.001–0.15 displays a slightly lower content of external T_2_ sites and lower benzene transport rate, indicating that the limited number and accessibility of external active sites are likely the primary reasons for the low catalytic activity of MWW-0.001–0.15. The primary products of the reaction were cyclohexylbenzene (CHB) and dicyclohexylbenzene (DCHB), with CHB being the more desirable product. As shown in Figure 9(c), the selectivity to CHB was notably higher over MWW-0.01–0.15 (86.3%) and MWW-0.05–0.15 (86.6%) than over MCM-22 (84.8%). This indicates that CHB diffuses more readily through the MWW-*x*-0.15 samples, minimizing consecutive alkylation reactions and undesirable side reactions compared to the conventional MCM-22 catalyst. These results highlight that MWW-*x*-0.15 catalysts provide more external active T_2_ sites and higher benzene diffusion rates with higher density of accessible external surface acid sites, which are essential for efficient benzene alkylation reactions. In summary, MWW-0.01–0.15 and MWW-0.05–0.15 demonstrate superior catalytic performance in the benzene alkylation reaction compared to MCM-22. Furthermore, the MWW-*x*-0.15 zeolites synthesized with minimal amounts of TMAdaOH and environmentally friendly, cost-effective cyclohexylamine offer promising potential for practical applications in industrial catalysis.

**Figure 9:**
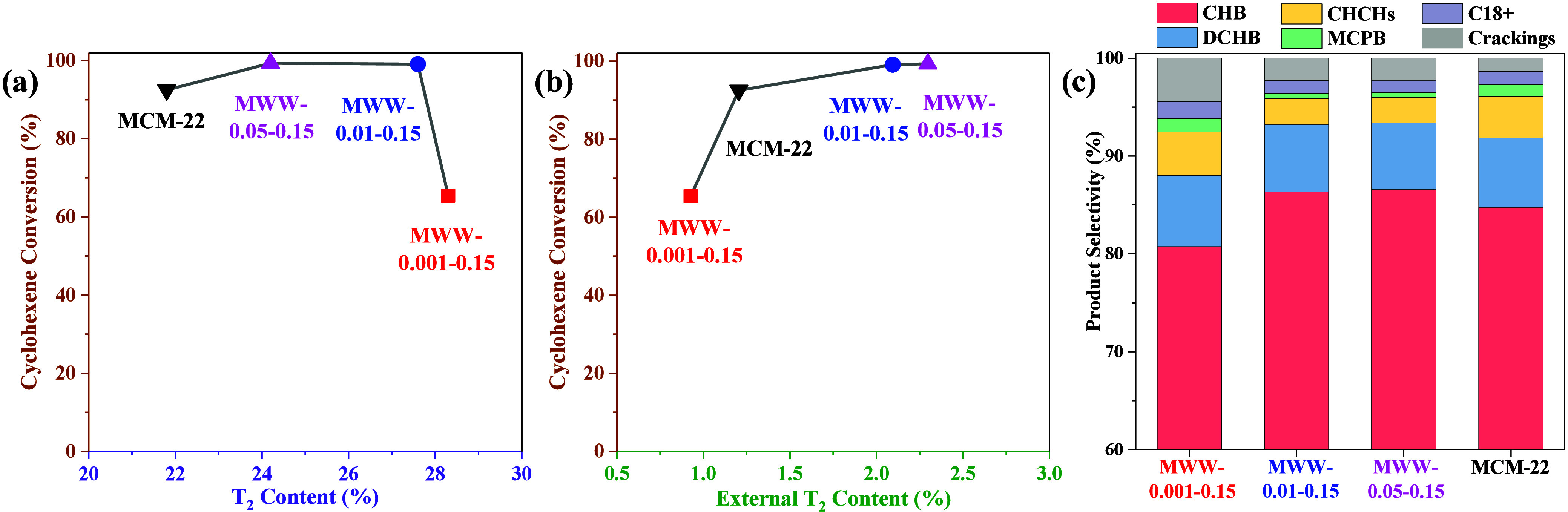
Catalytic performance of benzene alkylation with cyclohexene over H-form MWW zeolites produced with varying amounts of TMAdaOH and conventional MCM-22. (a) Relationship between cyclohexene conversion and T_2_ content; (b) relationship between cyclohexene conversion and external T_2_ content, and (c) product selectivity.

## 4 Conclusions

In conclusion, MWW zeolites with tunable aluminum distributions and enriched external acid sites were successfully synthesized through the cooperative use of TMAdaOH and cyclohexylamine as dual OSDAs. The synthesis temperature, time and Na^+^ were identified as critical parameters for achieving highly crystalline MWW zeolites. Systematic characterizations, especially advanced solid-state NMR, revealed the roles of the dual OSDAs in directing the MWW structure and modulating the distribution of aluminum sites. With the presences of both TMAdaOH and cyclohexylamine, TMAda^+^ preferentially stabilizes 12-*ring* channels (the supercages and external surface semi-supercages), while cyclohexylamine stabilizes both 12-*ring* channels and intralayer 10-*ring* channels. The introduction of TMAdaOH facilitated the formation of the MWW structure, and weakened the quaternization of cyclohexylamine, which in turn adjusted the distributions of aluminum sites. The proportion of total T_2_ aluminum sites decreased with the increase of TMAdaOH content, but the concentrations of T_2_ aluminum sites located in the external surface semi-supercages increased, together with the external surface area and benzene diffusion rates. The improved exposed T_2_ sites and facilitated reactant (benzene) diffusion properties predominately contribute to the activities in the cracking of TiPB and the alkylation of benzene with cyclohexene. This study illustrated the importance of Al distribution and that of the accessibility of the active acid sites. The reason for zeolites with enriched specific active Al sites showing comparable or poor catalytic performance is that the so-called “active sites” are actually not accessible to reactants. To some extent, the delaminated MWW type zeolites with large external surfaces reported before displayed higher activities because of the structure feature of the MWW type framework that the locations of T_2_ sites are in the supercages and the “broken” supercages (surface semi-supercages). This study provides valuable insights into the design and synthesis of MWW zeolite with tunable external acid sites using small amounts of low-toxicity OSDAs. The findings highlight the promising application potential of these MWW zeolites in industrial catalysis.

## Supplementary Material



## Notes

Further characterization and measurements of samples, including XRD, SEM, N_2_ adsorption–desorption, textural properties, NH_3_-TPD, IR, NMR and catalytic performance of the cracking of TiPB reaction (PDF)
