# Supplementary material for: Modulation of Al Sites in MWW Zeolites with Enhanced Catalytic Performance by Dual Organic Structure-Directing Agents
Source: Chem Bio Eng. 2025 Apr 10;2(6):358–69. doi: 10.1021/cbe.5c00016 (PMC12207279; doi:10.1021/cbe.5c00016)
Supplement: Supplementary file 1 [file be5c00016_si_001.pdf]

Supporting Information

Modulation of Al Sites in MWW Zeolites with Enhanced Catalytic Performance by Dual Organic Structure-Directing Agents

Chuang Liu[a,b,+], Guodong Qi[c,+], Yudan Gong[b], Darui Wang[b], Wenhua Fu[b], Fang Liu[b], Jun Xu[c]*, Dianhua Liu[a]*, Zhendong Wang[b]* and Weimin Yang[a,b]*

[a] School of Chemical Engineering, East China University of Science and Technology, Shanghai, 200237, China

[b] State Key Laboratory of Green Chemical Engineering and Industrial Catalysis, SINOPEC Shanghai Research Institute of Petrochemical Technology Co., Ltd., Shanghai, 201208, China

[c] National Center for Magnetic Resonance in Wuhan, State Key Laboratory of Magnetic Resonance Spectroscopy and Imaging, Innovation Academy for Precision Measurement Science and Technology, Chinese Academy of Sciences, Wuhan 430071, China

[+] The authors contribute equally to this work

* Corresponding author. Tel: +86-21-68466427; E-mail: yangwm.sshy@sinopec.com;

* Corresponding author. Tel: +86-21-68461267; E-mail: wangzd.sshy@sinopec.com;

* Corresponding author. Tel: +86-21-64252694; E-mail: dhliu@ecust.edu.cn;

* Corresponding author. Tel: +86-27-87197359; E-mail: xujun@wipm.ac.cn.

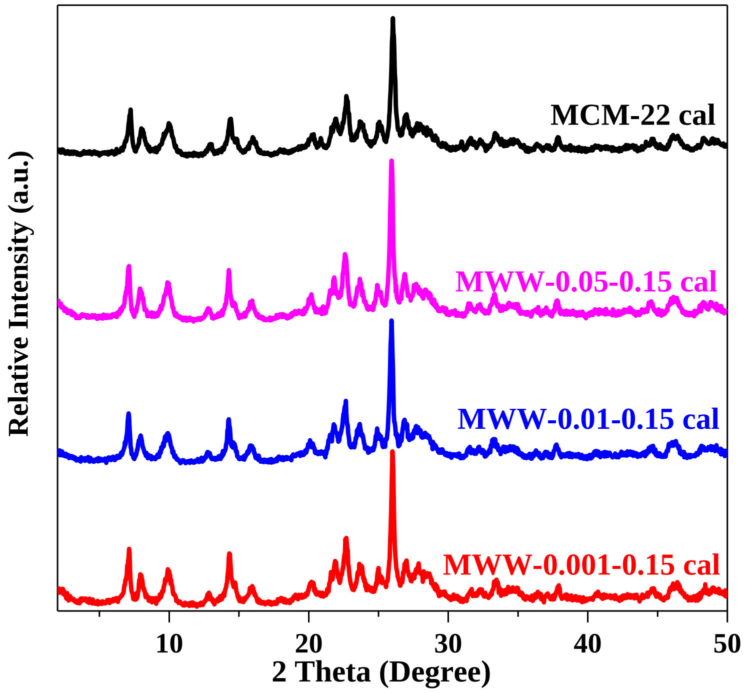

Figure S1. XRD patterns of calcined **MWW** zeolites synthesized with varying amounts of TMAdaOH and conventional MCM-22.

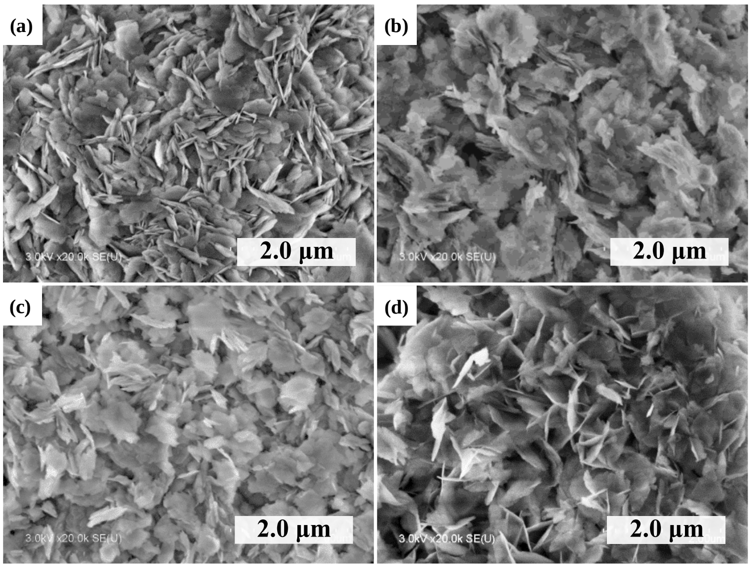

Figure S2. SEM images of (a-c) MWW zeolites produced with varying amounts of TMAdaOH (0.001, 0.01 and 0.05) and (d) conventional MCM-22 zeolite.

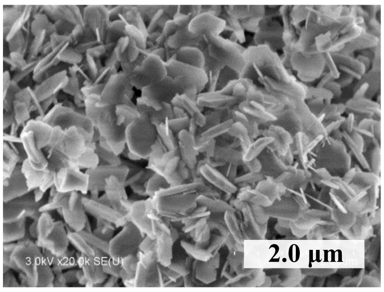

Figure S3. SEM image of FER-0-0.15 zeolite produced without adding TMAdaOH.

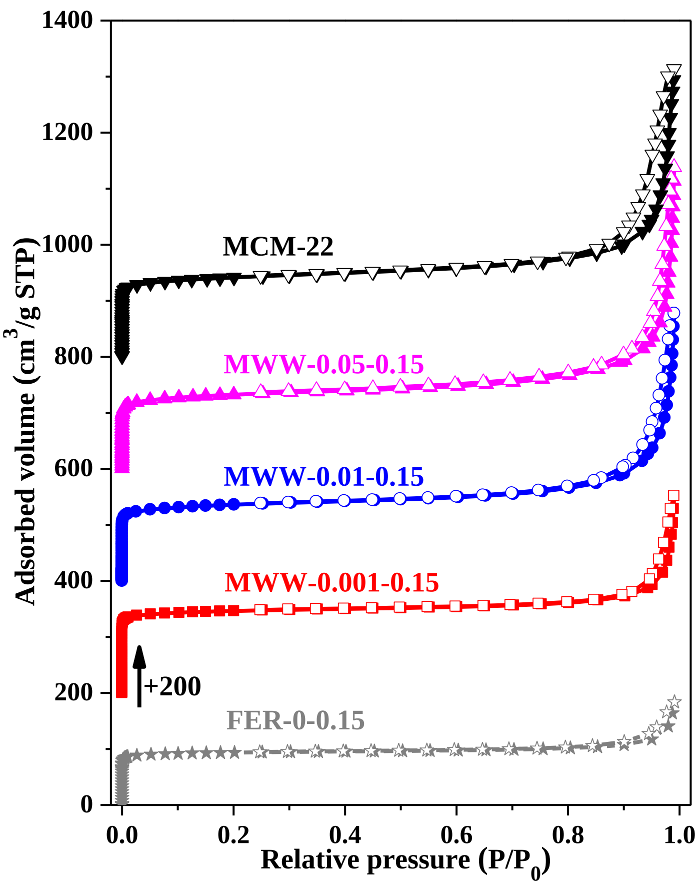

Figure S4. N_2 adsorption-desorption isotherms of MWW zeolites produced with varying amounts of TMAdaOH and conventional MCM-22 zeolite.

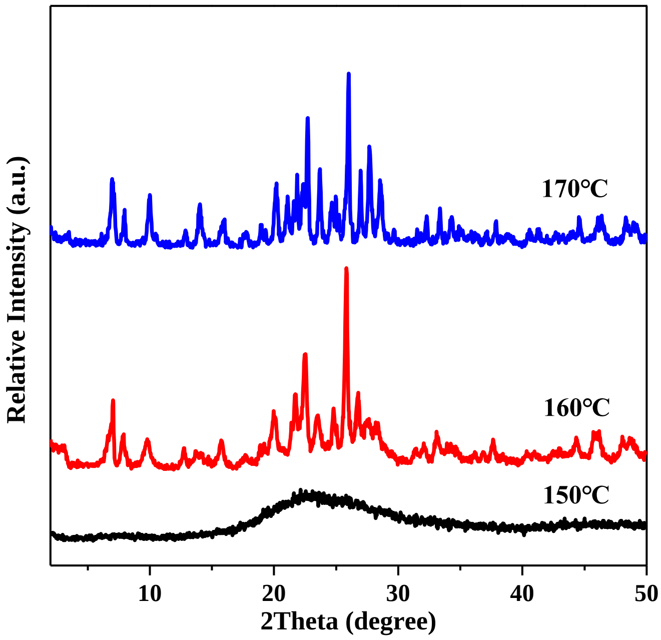

Figure S5. XRD patterns of samples synthesized with varying temperatures.

Synthesis conditions: molar composition of gel is 1 SiO_2: 0.033 Al_2O_3: 0.01 TMAdaOH: 0.15 cyclohexylamine: 0.12 NaOH: 18 H_2O, crystallized for 3 days.

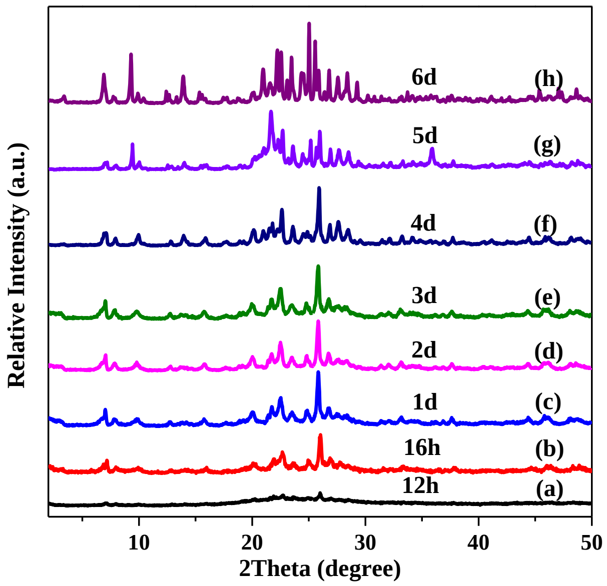

Figure S6. XRD patterns of samples synthesized with varying synthesis times.

Synthesis conditions: molar composition of gel is 1 SiO_2: 0.033 Al_2O_3: 0.01 TMAdaOH: 0.15 cyclohexylamine: 0.12 NaOH: 18 H_2O, crystallized at 160 °C.

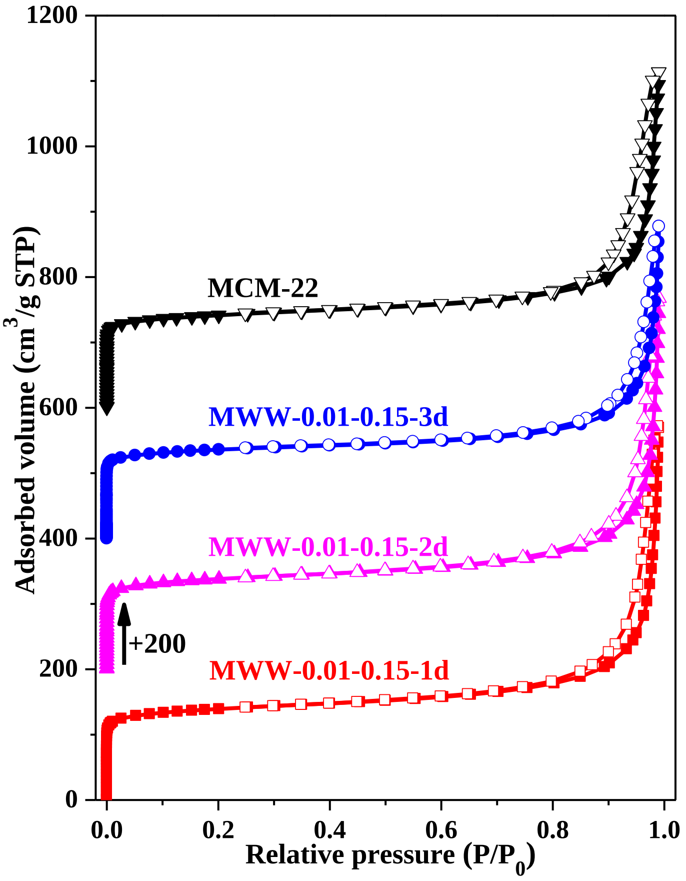

Figure S7. N_2 adsorption-desorption isotherms of MWW zeolites synthesized with varying synthesis times and conventional MCM-22.

Synthesis conditions: molar composition of gel is 1 SiO_2: 0.033 Al_2O_3: 0.01 TMAdaOH: 0.15 cyclohexylamine: 0.12 NaOH: 18 H_2O, crystallized at 160 °C.

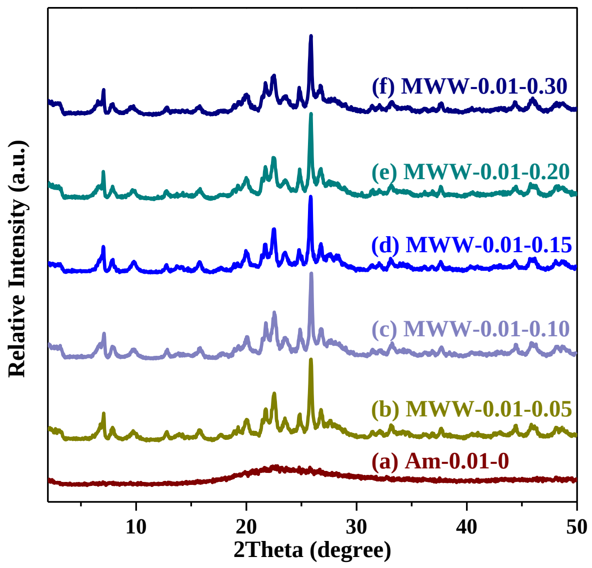

Figure S8. XRD patterns of samples synthesized with varying amounts of cyclohexylamine; (a) Am-0.01-0, (b) MWW-0.01-0.05, (c) MWW-0.01-0.10, (d) MWW-0.01-0.15, (e) MWW-0.01-0.20, (f) MWW-0.01-0.30.

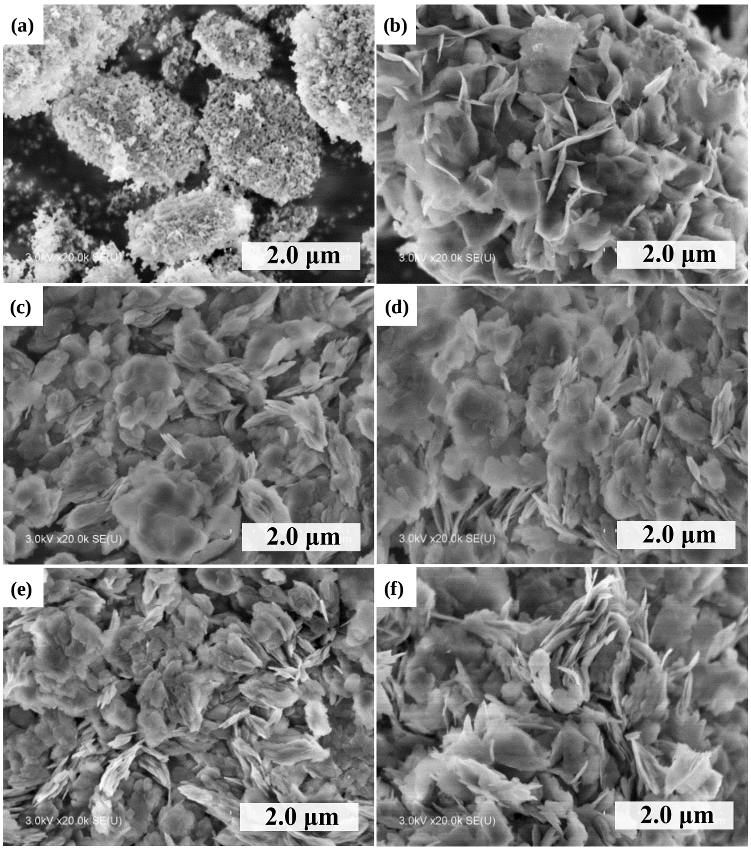

Figure S9. SEM images of samples synthesized with varying amounts of cyclohexylamine; (a) Am-0.01-0, (b) MWW-0.01-0.05, (c) MWW-0.01-0.10, (d) MWW-0.01-0.15, (e) MWW-0.01-0.20, (f) MWW-0.01-0.30.

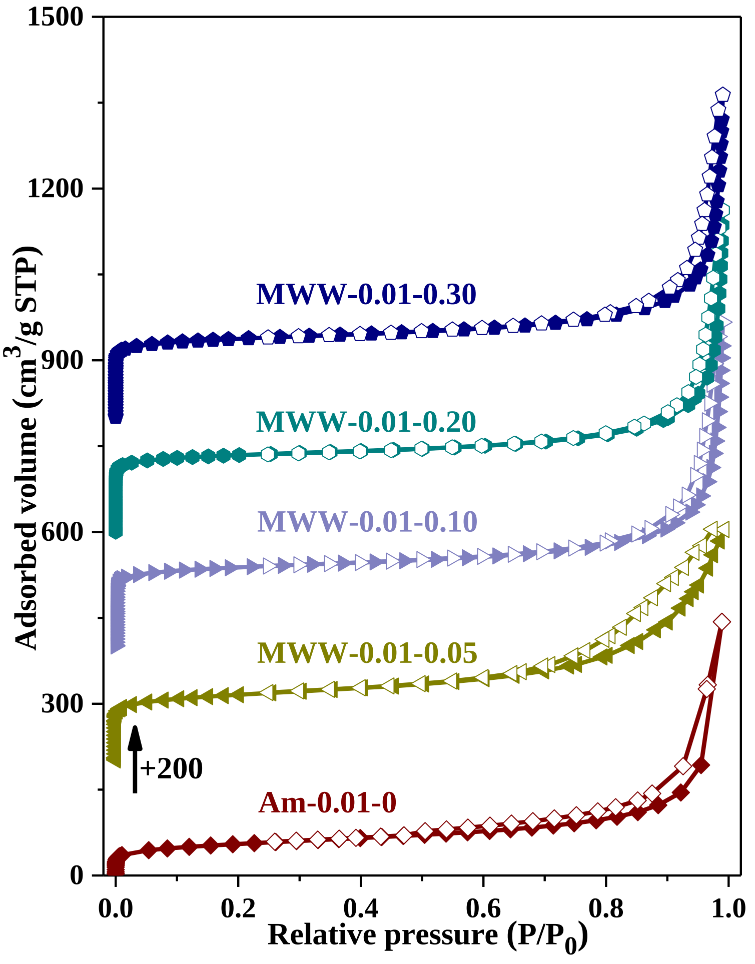

Figure S10. N_2 adsorption-desorption isotherms of the samples synthesized with varying amounts of cyclohexylamine.

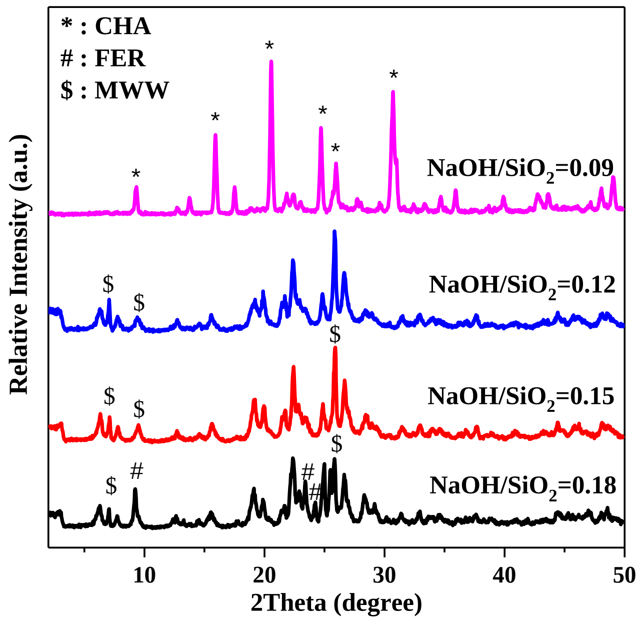

Figure S11. XRD patterns of samples synthesized with varying amounts of NaOH.

Synthesis conditions: molar composition of gel is 1 SiO_2: 0.033 Al_2O_3: 0.05 TMAdaOH: 0.15 cyclohexylamine: 0.09 - 0.18 NaOH: 18 H_2O, crystallized at 160 °C for 3 days.

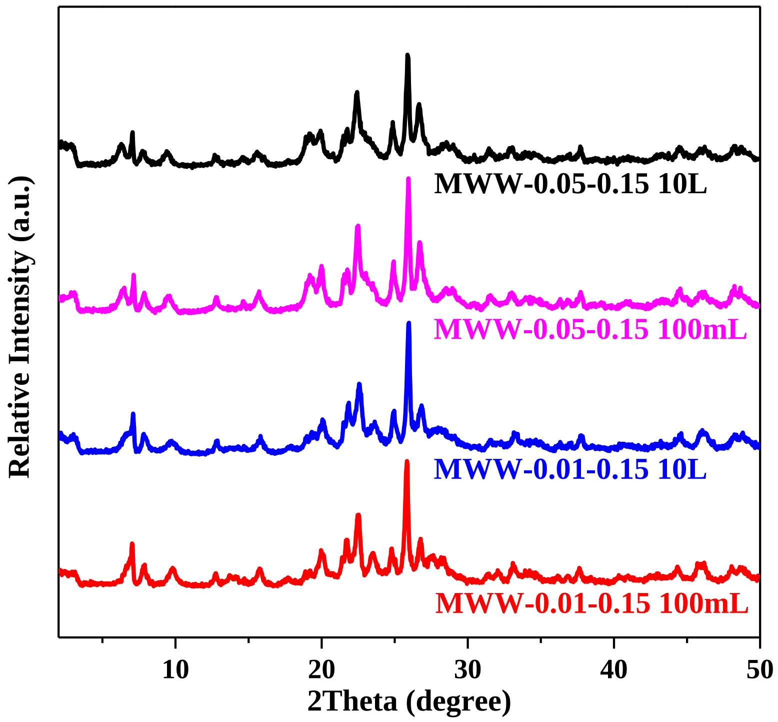

Figure S12. XRD patterns of the MWW zeolites synthesized with different scales.

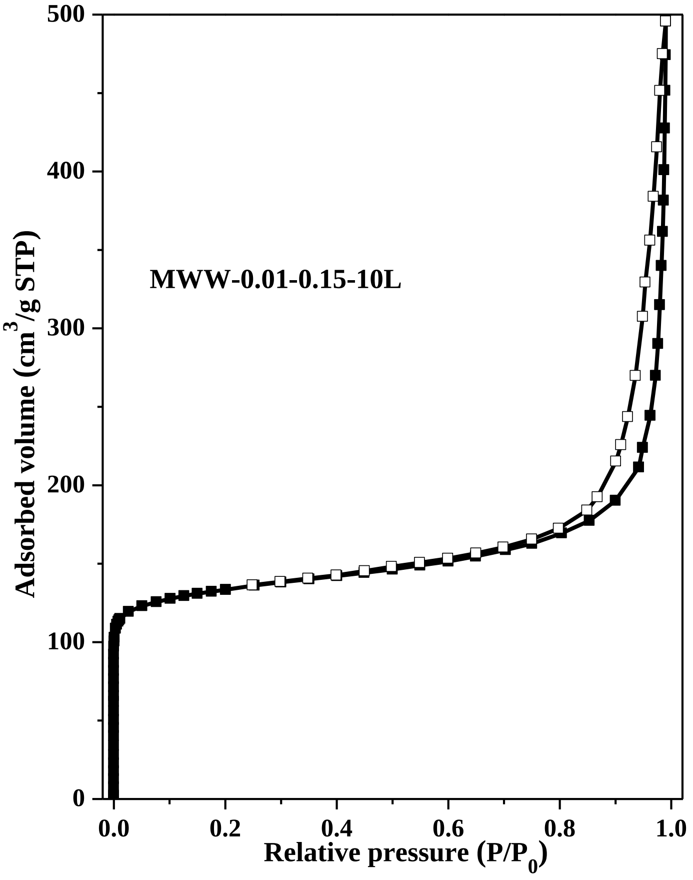

Figure S13. N_2 adsorption-desorption isotherm MWW-0.01-0.15 zeolite synthesized in a 10 L scale.

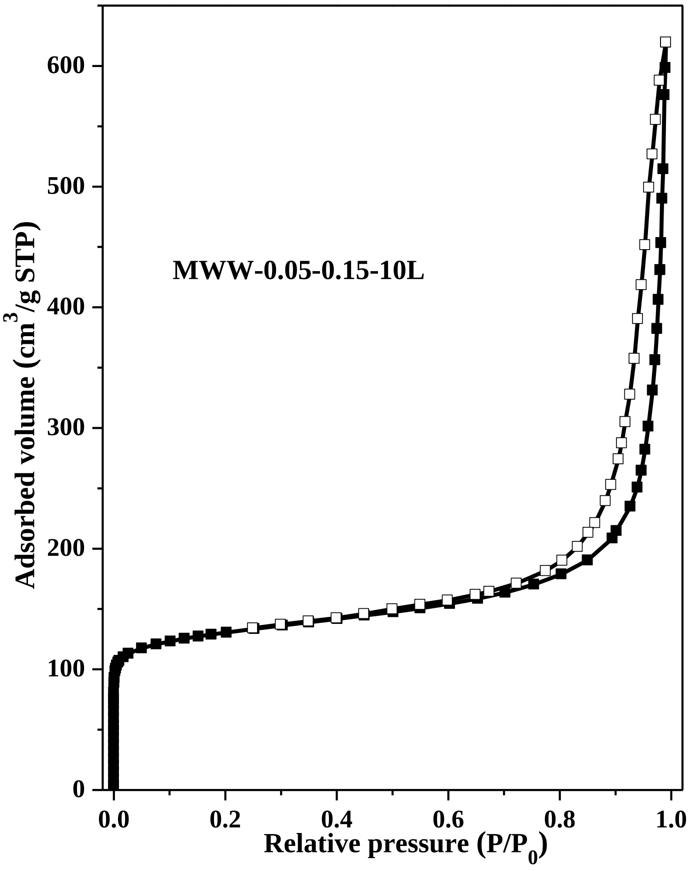

Figure S14. N_2 adsorption-desorption isotherm MWW-0.05-0.15 zeolite synthesized in a 10 L scale.

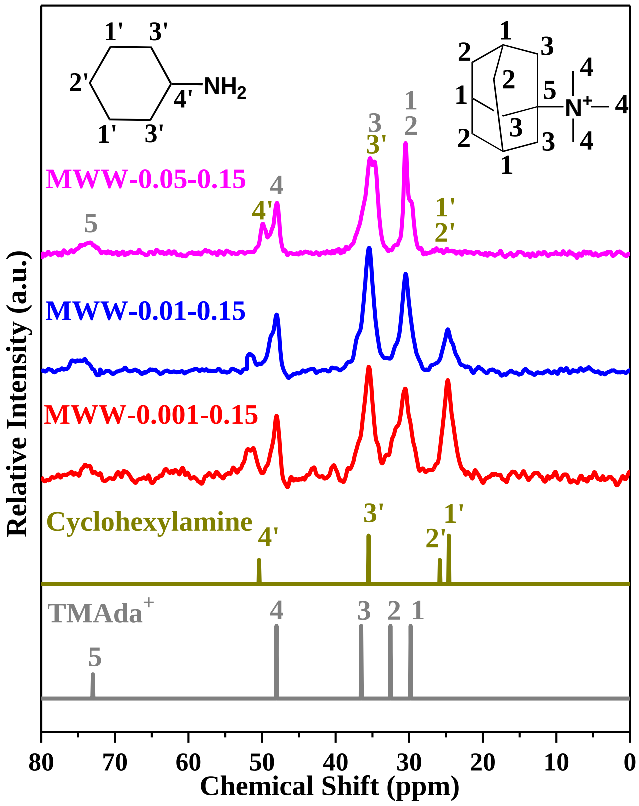

Figure S15. ^{1}H-^{13}C CP MAS NMR profiles of MWW zeolites synthesized with varying amounts of TMAdaOH.

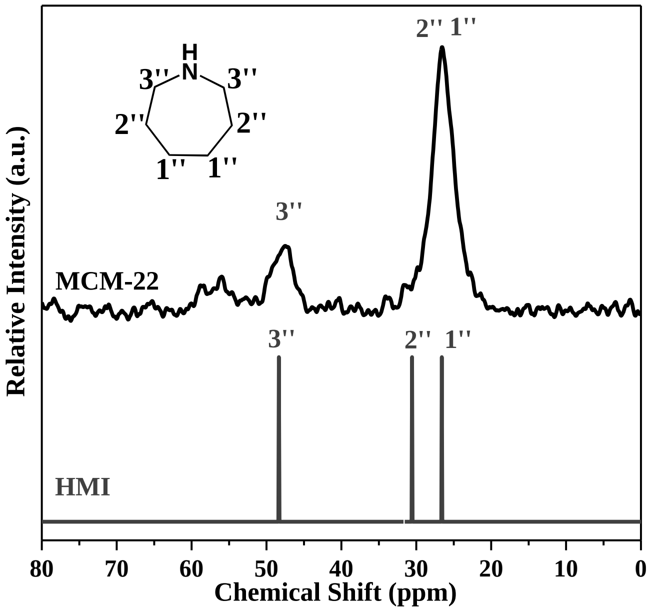

Figure S16. ^{1}H-^{13}C CP MAS NMR profiles of the conventional MCM-22 zeolite synthesized by HMI as OSDA.

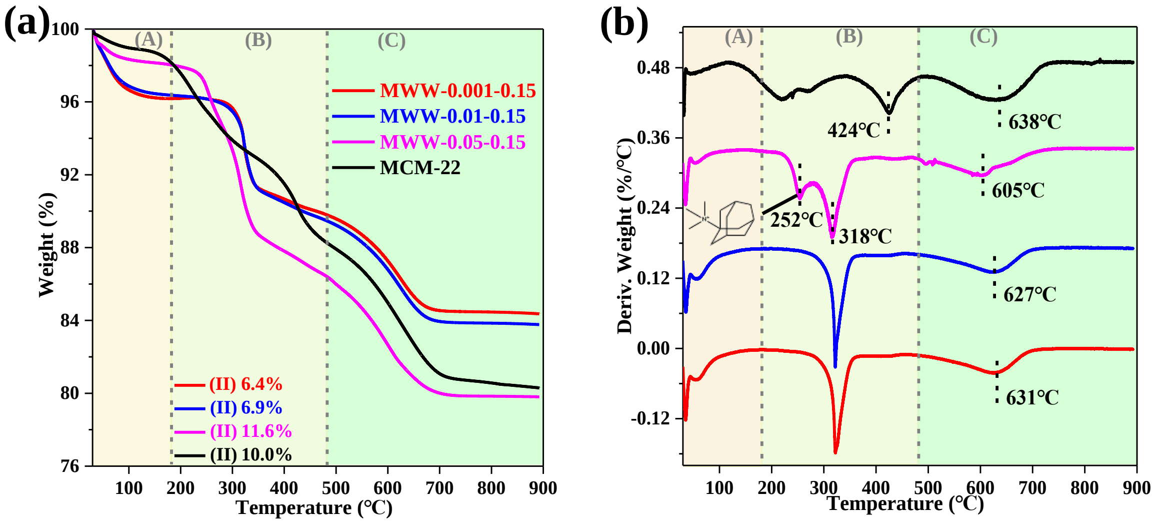

Figure S17. (a) TG and (b) DTG profiles of MWW zeolites produced with varying amounts of TMAdaOH and conventional MCM-22.

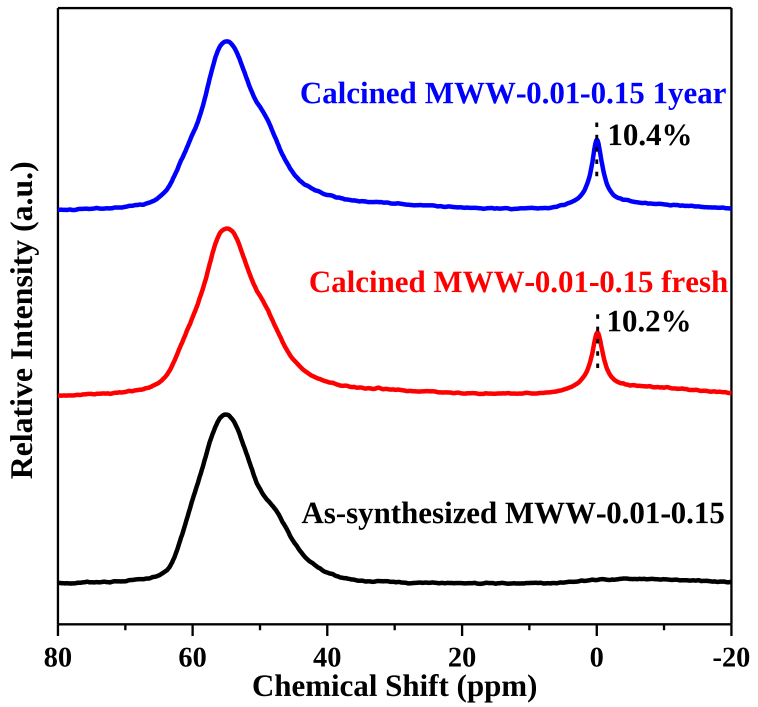

Figure S18. ^{27}Al MAS NMR profiles of as-syntheisized, freshly calcined and calcined (~1 year ago) MWW-0.01-0.15 zeolites.

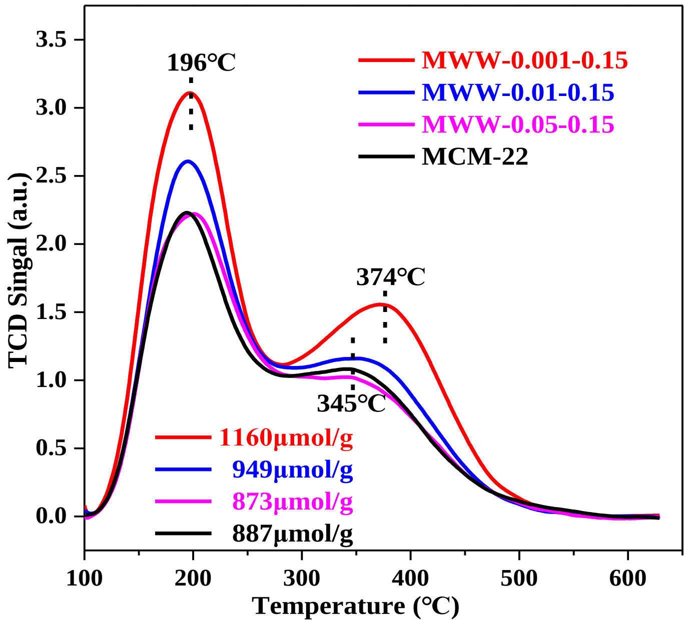

Figure S19. NH_3-TPD curves of the MWW zeolites synthesized with varying amounts of TMAdaOH and the MCM-22 zeolite.

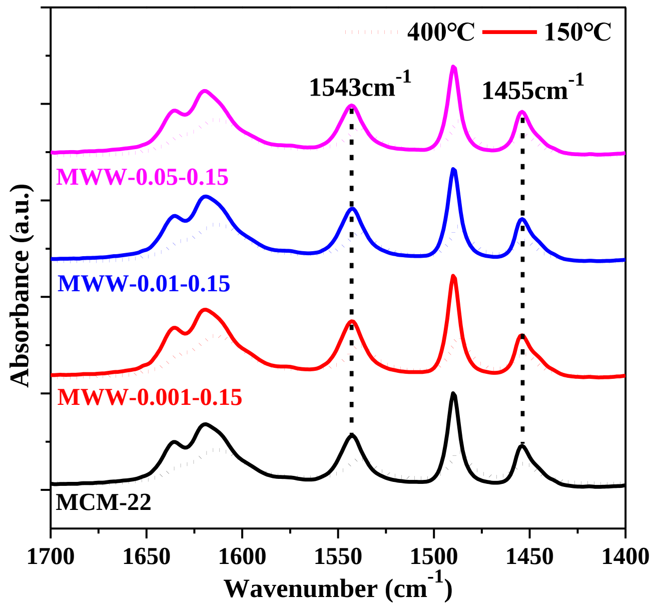

Figure S20. Py-IR spectra of MWW zeolites synthesized with varying amounts of TMAdaOH and MCM-22 zeolite.

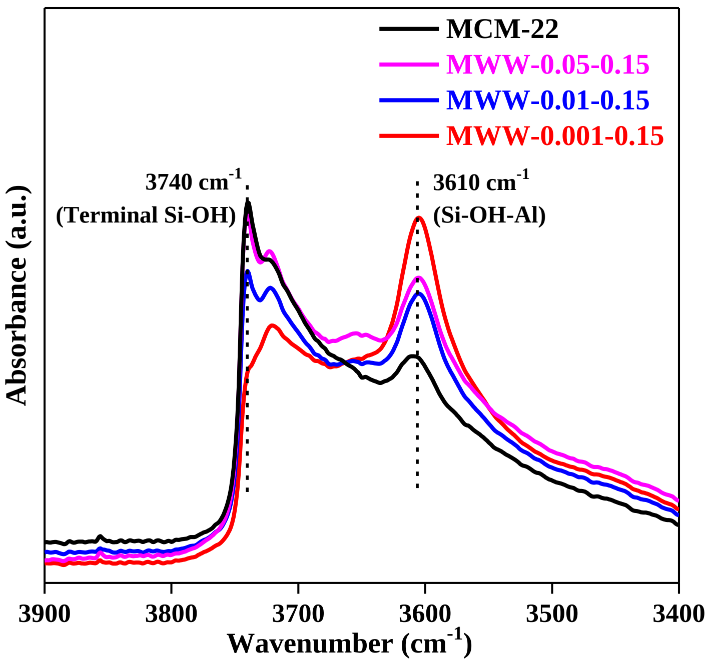

Figure S21. IR spectra in the OH-stretching region of MWW zeolites synthesized with varying amounts of TMAdaOH and MCM-22 zeolite.

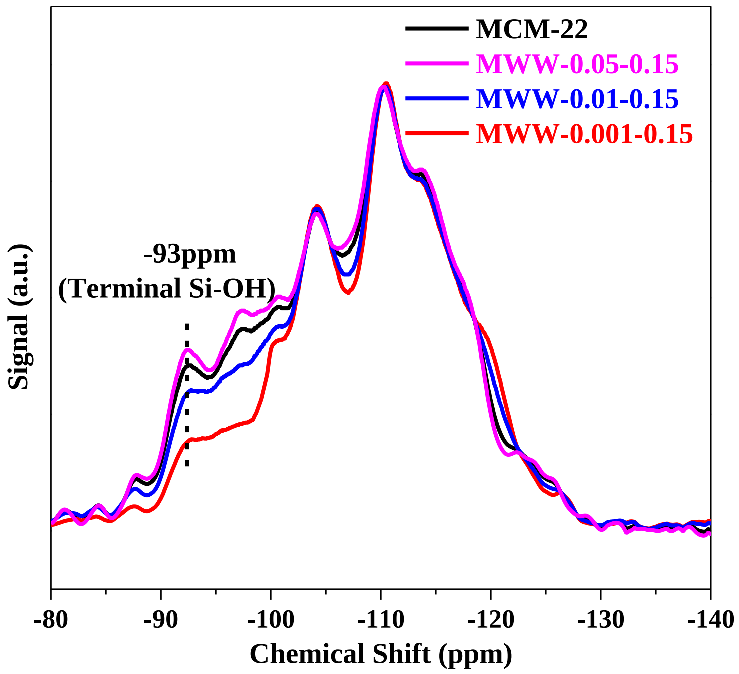

Figure S22. ^{29}Si NMR spectra of MWW zeolites synthesized with varying amounts of TMAdaOH and MCM-22 zeolite.

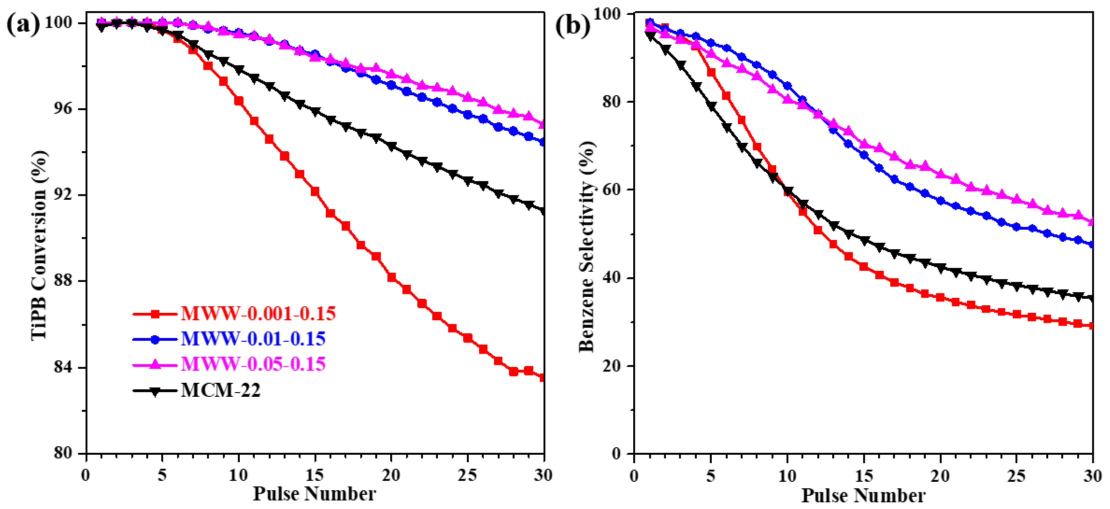

Figure S23. Catalytic performance of TiPB cracking over H-form MWW zeolites; (a) TiPB Conversion, (b) Benzene Selectivity.

Table S1. Textural properties samples synthesized with varying conditions.

Sample	S_{BET} (m^2/g)	S_{Ext} (m^2/g)	V_{Total} (cm^3/g)	V_{Micro} (cm^3/g)
FER-0-0.15	360	21	0.28	0.14
MWW-0.001-0.15	571	32	0.54	0.21
MWW-0.01-0.15	522	114	0.73	0.17
MWW-0.05-0.15	459	153	0.79	0.15
Am-0.01-0	169	89	0.66	0.03
MWW-0.01-0.05	476	155	0.57	0.09
MWW-0.01-0.10	497	136	0.74	0.16
MWW-0.01-0.20	513	119	0.74	0.17
MWW-0.01-0.30	534	108	0.80	0.17
MCM-22	510	102	0.74	0.17
MWW-0.01-0.15-1d	479	129	0.76	0.15
MWW-0.01-0.15-2d	496	120	0.76	0.16
MWW-0.01-0.15-10L	497	109	0.74	0.17
MWW-0.05-0.15-10L	478	139	0.92	0.15

Table S2. Weight loss of MWW zeolites at different temperature ranges.

Sample	Stage (A) (%)	Stage (B) (%)	Stage (C) (%)	Total weight loss (%)
MWW-0.001-0.15	3.8	6.4	5.4	15.6
MWW-0.01-0.15	3.6	6.9	5.7	16.2
MWW-0.05-0.15	1.9	11.6	6.6	20.1
MCM-22	1.8	10.0	8.0	19.8

Table S3. Acid properties of MWW zeolites synthesized with varying amounts of TMAdaOH and conventional MCM-22.

Sample	$B_{150°C}$ (μmol/g)[a]	$L_{150°C}$ (μmol/g)[a]	$B_{400°C}$ (μmol/g)[b]	$L_{400°C}$ (μmol/g)[b]
MWW-0.001-0.15	560	284	156	112
MWW-0.01-0.15	552	286	147	106
MWW-0.05-0.15	551	293	139	103
MCM-22	537	290	132	103

[a] & [b] Calculated from Py-IR spectra (B acid sites at 1543 cm^{-1}, L acid sites at 1455 cm^{-1}), [a] desorption at 150 °C, and [b] desorption at 400 °C.
